# Regulation of N-degron recognin-mediated autophagy by the SARS-CoV-2 PLpro ubiquitin deconjugase

**DOI:** 10.1080/15548627.2024.2442849

**Published:** 2024-12-26

**Authors:** Carlos Ayala-Torres, Jiangnan Liu, Nico P. Dantuma, Maria G. Masucci

**Affiliations:** Department of Cell and Molecular Biology, Karolinska Institutet, Solna, Sweden

**Keywords:** HSPA5/BiP/GRP78, N-degron, PLpro, reticulophagy, SARS-CoV-2, SQSTM1/p62

## Abstract

Viral proteases play critical roles in the host cell and immune remodeling that allows virus production. The severe acute respiratory syndrome coronavirus-2 (SARS-CoV-2) papain-like protease (PLpro) encoded in the large nonstructural protein 3 (Nsp3) also possesses isopeptidase activity with specificity for ubiquitin and ISG15 conjugates. Here, we interrogated the cellular interactome of the SARS-CoV-2 PLpro catalytic domain to gain insight into the putative substrates and cellular functions affected by the viral deubiquitinase. PLpro was detected in protein complexes that control multiple ubiquitin and ubiquitin-like (UbL) regulated signaling and effector pathways. By restricting the analysis to cytosolic and membrane-associated ubiquitin ligases, we found that PLpro interacts with N-recognin ubiquitin ligases and preferentially rescues type I N-degron substrates from proteasomal degradation. PLpro stabilized N-degron carrying HSPA5/BiP/GRP78, which is arginylated in the cytosol upon release from the endoplasmic reticulum (ER) during ER stress, and enhanced the Arg-HSPA5-driven oligomerization of the N-recognin SQSTM1/p62 that serves as a platform for phagophore assembly. However, while in addition to Arg-HSPA5 and SQSTM1/p62, ATG9A, WIPI2, and BECN1/Beclin 1 were detected in PLpro immunoprecipitates, other components of the autophagosome biogenesis machinery, such as the ATG12–ATG5-ATG16L1 complex and MAP1LC3/LC3 were absent, which correlated with proteolytic inactivation of ULK1, impaired production of lipidated LC3-II, and inhibition of reticulophagy. The findings highlight a novel mechanism by which, through the reprogramming of autophagy, the PLpro deubiquitinase may contribute to the remodeling of intracellular membranes in coronavirus-infected cells.

## Introduction

Since the emergence of Severe Acute Respiratory Syndrome Coronavirus-2 (SARS-CoV-2) and the associated Coronavirus Disease 2019 (COVID-19), extraordinary efforts in pharmaceutical research have led to the rapid development of preventive vaccines, neutralizing monoclonal antibodies, and small molecule drugs [1,2]. Still, much remains to be learned about the viral proteins, their cellular targets, mechanisms of action, and how their function contributes to viral pathogenesis.

The genome of SARS-CoV-2 encodes 16 nonstructural proteins (Nsps) that play different roles in the virus life cycle [3]. The largest Nsp, Nsp3, is a key component of the replication and transcription complex/RTC that assembles on host cell membranes where the production of new viral genomes occurs [4–6]. Encoded within Nsp3 is a papain-like protease (PLpro) that cleaves the viral polyprotein at LXGG consensus sites, releasing Nsp1, Nsp2, and Nsp3 [7]. The release of Nsp3 activates the main viral protease (3CLpro) that completes the processing of the viral polyprotein into the subunits of the viral polymerase [8]. The structure of the PLpro domains of coronavirus Nsp3s resembles that of human ubiquitin-specific proteases (deubiquitinases, DUBs), and the consensus cleavage site is also the target sequence recognized by many deubiquitinases [9]. Thus, in addition to the endopeptidase activity, the viral enzymes PLpro possess an isopeptidase activity that targets substrates post-translationally modified by conjugation with ubiquitin or the ubiquitin-like protein ISG15 (ISG15 ubiquitin like modifier) [10,11].

Substrate modification by ubiquitin and ubiquitin-like molecules (UbLs), including ISG15, SUMO (small ubiquitin like modifier), NEDD8 (NEDD8 ubiquitin like modifier), and UFM1 (ubiquitin fold modifier 1) [12], involves an enzymatic cascade, starting with an activating enzyme E1, followed by a conjugating enzyme E2 and by a ligase E3, which typically forms an isopeptide bond between the carboxyl terminus of the modifier and the ε-amino group of a lysine residue on the target protein [13]. In addition, the intensity and duration of signaling are regulated by isopeptidases that reverse the reaction [14,15]. Posttranslational modifications by ubiquitin and UbLs play a pivotal role in the regulation of a wide variety of cellular functions that are captured by viruses to promote the establishment of an environment conducive to virus replication while counteracting the host antiviral defenses [16–21]. The best-understood strategies involve the viral protein-mediated inactivation or repurposing of cellular enzymes, which, together with the expression of viral-encoded functional homologs, contributes to the extensive host cell and immune remodeling that allows efficient production and spread of infectious virus. The viral enzymes often differ from their cellular counterparts in structure, specificity, and mode of action, making them attractive candidates for the development of specific antivirals [22,23].

Early studies on the PLpro encoded by SARS-CoV-2 revealed that, different from the homologs encoded by other coronaviruses, the isolated catalytic domain exhibits a strong preference for ISG15 conjugates due to distinctive interactions with the amino-terminal ubiquitin-like domain of ISG15 [24,25]. ISG15 plays multiple roles in the host antiviral responses [26], including inhibition of viral capsid assembly and virus budding via conjugation to viral proteins [27–29] and upregulation of innate immune responses via conjugation to various cellular immune signaling and effector proteins [30]. Thus, the capacity of SARS-CoV-2 PLpro to antagonize ISGylation was shown to attenuate the type I IFN response via de-ISGylation of the IRF3 (interferon regulatory factor 3) [24] and the viral RNA sensor IFIH1/MDA5 (interferon induced with helicase C domain 1) [31]. As ubiquitin deconjugases, the PLpro enzymes of SARS coronaviruses preferentially hydrolyze Lys48-linked ubiquitin chains via a mechanism that relies on the presence of two ubiquitin-binding sites, where the occupation of the distal site is most important for poly-Ub processing [32,33]. Several coronavirus deubiquitinases, including SARS-CoV-2 PLpro, were shown to regulate innate immune responses via deubiquitination of STING1 (stimulator of interferon response cGAMP interactor 1) and the destabilization of STING1 complexes [34,35], but very little is known on other substrates or signaling pathways targeted by the viral enzyme. Notably, due to the large size of Nsp3, most studies aiming to characterize the function of PLpro have examined the minimal catalytic domain, which does not account for the possible targeting and regulatory effect of other Nsp3 domains. Indeed, although the isolated PLpro domain and the membrane-bound multidomain Nsp3 have largely overlapping interactomes, Nsp3 was shown to be a more active protease, cleaving ISG15 and K48-linked ubiquitin chains with greater efficiency [36], which highlights the contribution of elements outside of the minimal PLpro domain in regulating the enzymatic function in infected cells.

In this investigation, we aimed to gain new insight into the cellular substrates of the deubiquitinase activity of SARS-CoV-2 PLpro. Starting from the hypothesis that in infected cells, the membrane-associated viral enzyme regulates events occurring at or in the vicinity of cellular membranes, we searched the PLpro interactome for ubiquitin ligases that localize prevalently in the cytosol, the endoplasmic reticulum (ER), or Golgi. We found that PLpro interacts with N-recognin ubiquitin ligases and selectively stabilizes N-degron substrates, such as the signal sequence deleted species of the ER-chaperone HSPA5 that is arginylated upon release in the cytosol during ER stress. Catalytically active PLpro enhanced the R-HSPA5-driven oligomerization of SQSTM1/p62 and promoted the selective recruitment of certain components of autophagosome biogenesis machinery while inhibiting the accumulation of lipidated LC3 and impairing reticulophagy, pointing to an important role of the deubiquitinase in the remodeling of ER membranes during viral infection.

## Results

### PLpro interacts with N-degron E3 ligases

To achieve an unbiased overview of the SARS-CoV-2 PLpro interactome and to identify putative substrates, FLAG-tagged versions of the catalytically active PLpro domain (FLAG-PLpro, strain MN90894.3 aa 746–1060), or a catalytic mutant where the Cys111 residue (Cys857 of the full-length Nsp3) was mutated to Ala (FLAG-PLpro^mut^) (Figures S1A, and S1B) were transfected in U2OS cells along with a FLAG-empty vector (FLAG-ev), and interacting proteins were immunoprecipitated using anti-FLAG conjugated agarose beads. The immunoprecipitates were fractionated by SDS-PAGE, and four gel slices obtained from two independent immunoprecipitation assays performed in duplicate were subjected to tandem mass spectrometry (Figure S2A and S2B). Three hundred two putative interactors identified by two or more unique spectral counts in at least three of the four samples, and either absent in the FLAG-ev immunoprecipitations or showing Log_2_ enrichment ≥ 2, were detected in the immunoprecipitates of cells expressing FLAG-PLpro. Of these, 40 were also detected in immunoprecipitates of cells expressing FLAG-PLpro^mut^, while 11 were detected only in immunoprecipitates of the catalytic mutant PLpro. Based on Gene Ontology annotations, the interactome identified by the sum of PLpro and PLpro^mut^ interactors is enriched in proteins involved in ribosome biogenesis, RNA metabolic processes, mRNA translation nuclear-cytoplasmic transport, vesicular trafficking, and ER stress responses and includes several members of the ubiquitin-proteasome system/UPS, such as proteasome subunits, ubiquitin ligases, and deconjugases (Figure S2A). Analysis of the protein interaction network identified a major interaction hub centering around ribosome biogenesis, including ribosome proteins and chaperones, mRNA translation, including several subunits of the pre-initiation and initiation complexes, the ER-ribosome quality control/ER-RQC, and ER-stress responses (Figure S2B). These findings confirm and extend previously reported mass spectrometry data [37–40], pointing to an important role of the viral enzyme in the host cell remodeling that allows productive infection.

**Figure 1.**
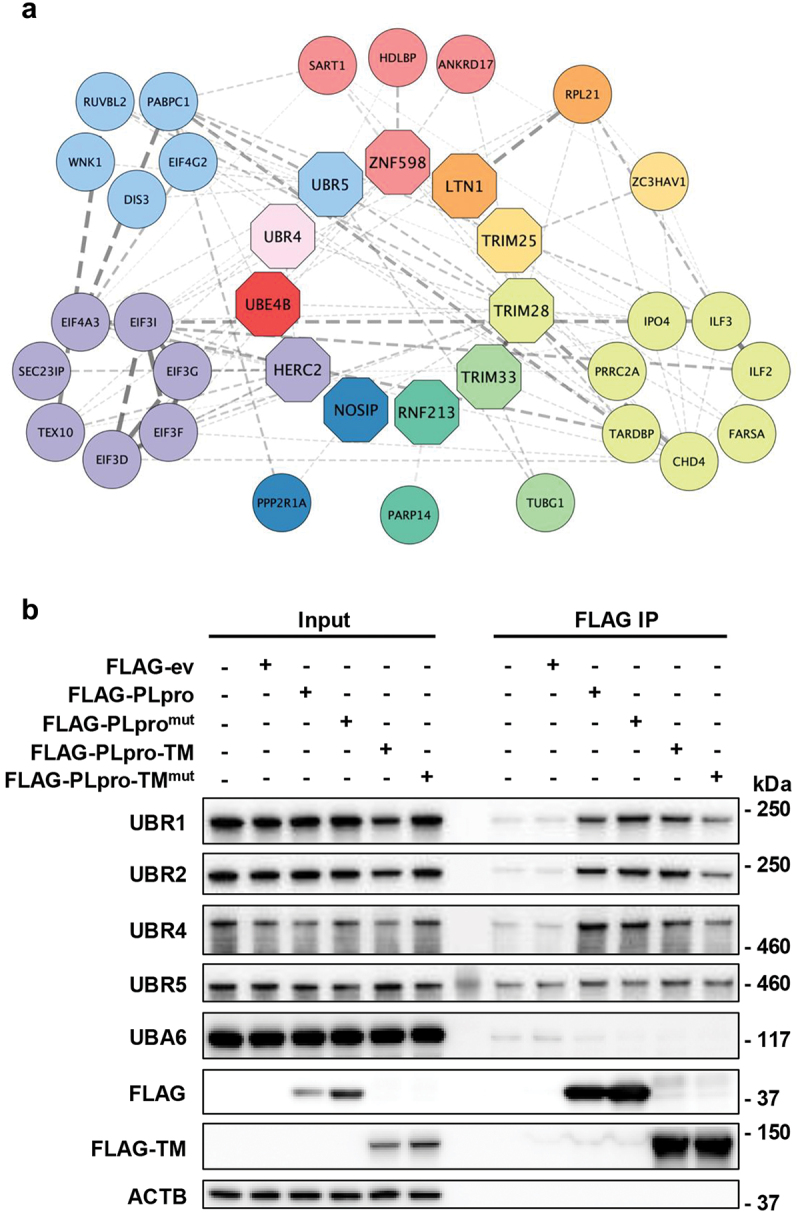
PLpro interacts with N-recognin ubiquitin ligases. (a) STRING network diagram of the PLpro-interacting cytosolic and membrane-associated ubiquitin ligases and the shared interacting partners identified by co-immunoprecipitation and mass spectrometry. Mass spectrometry analysis was performed on FLAG-tag immunoprecipitates of U2OS cells transiently transfected with FLAG-ev/PLpro/PLpro^mut^, and bona fide PLpro interactors were identified as described in methods. The PLpro-interacting ligases and shared interacting partners found in the interactome are color-coded. (b) Representative western blots that illustrate the interaction of PLpro with N-recognin ligases. Lysates of HEK293T cells transfected with plasmids expressing FLAG-ev/PLpro/PLpro^mut^ were immunoprecipitated with anti-FLAG coated beads, and western blots were probed with the indicated antibodies. β-actin served as a loading control. Each interaction was validated in at least two independent co-immunoprecipitation experiments.

In view of the coordinated activity of ubiquitin ligases and deconjugases and based on the assumption that, due to the ER-membrane localization of Nsp3, the primary targets of the viral enzyme would be either cytosolic or ER-associated proteins, we restricted our analysis to the interaction with cellular E3 ligases and the shared interacting partners that are prevalently found in the cytosol or are associated with the ER and Golgi membrane networks. Eleven E3 ligases matched this selection criteria (Figure 1A and Table S1). These included the ER-ribosome quality control ubiquitin ligases ZNF598 and LTN1 that mediate the recognition of stalled ribosomes and the proteasome-dependent degradation of aberrant translation products, respectively [41], the ubiquitin and ISG15 ligase TRIM25 that, via ubiquitination of RIGI, plays a key role in the regulation of type I IFN responses [42,43], the TRIM28, TRIM33, RNF213, NOSIP, and HERC2 ubiquitin ligases that participate in various signaling pathways that regulate inflammatory and antiviral responses [44–48], the E4 elongase UBE4B that extends Ub-chains initiated by several E3 ligase [49], and two N-recognin ligases, UBR4 and UBR5, that ubiquitinate substrates carrying N-terminal destabilization signals known as N-degrons [50]. The possible involvement of PLpro in N-degron-dependent events appeared particularly interesting since the pathway controls cellular functions that are involved in antiviral responses, including apoptosis [50] and autophagy [51,52], while the generation of N-degron substrates may be enhanced during SARS-CoV-2 infection through upregulation of the arginyl-tRNA-protein transferase ATE1 [53].

To explore the possibility that PLpro may regulate the N-degron pathway, we first sought to validate the interaction with the relevant E3 ligases by co-immunoprecipitation of the endogenous proteins in lysates of HEK293T and HeLa cells transfected with FLAG-PLpro, the catalytic mutant, FLAG-PLpro^mut^, or as control FLAG-ev. The cell lines were chosen for the validation studies due to consistently higher transfection efficiency. Two additional members of the N-recognin ligase family, UBR1 and UBR2, and the cognate E1 enzyme UBA6 were included in the analysis. To further assess whether the interaction may also occur in the context of the membrane-associated enzyme, a pair of catalytically active and inactive PLpro constructs that include the transmembrane and the C-terminal cytosolic domains (PLpro-TM, Figures S1A, and S1C) was also used as bait for the immunoprecipitation. UBR1, UBR2, and UBR4 were readily detected in the HEK293T immunoprecipitates of PLpro and PLpro-TM independently of catalytic activity, whereas UBA6 and ACTB/β-actin were absent (Figure 1B), confirming the specificity of the interaction and supporting the conclusion that the native PLpro may participate in protein complexes involved in N-degron-regulated processes. Of note, UBR5 appeared to be slightly enriched in the immunoprecipitates, but the specificity of the interaction with PLpro is uncertain due to the consistently high background observed in immunoprecipitates of the untransfected and FLAG-ev transfected controls (Figure 1B). Similar results were obtained when the co-immunoprecipitation assays were performed in lysates from transfected HeLa cells (Figure S2C).

### PLpro preferentially stabilizes N-degron substrates

**Figure 2.**
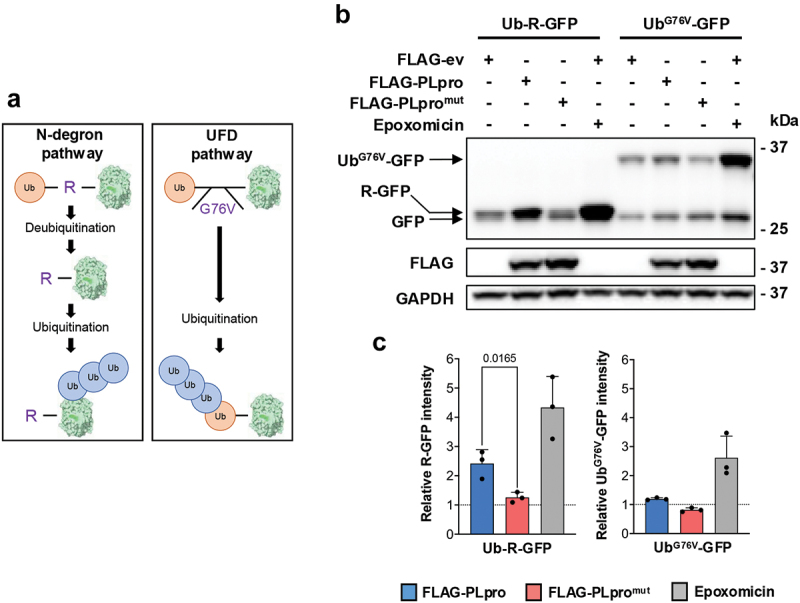
PLpro preferentially stabilizes N-degron substrates. (a) schematic illustration of the Ub-fusion reporters. The Ub-X-GFP reporters are cotranslationally cleaved by cellular ubiquitin deconjugases at the Ub-X junction, producing Ub and X-GFP. The Nt-Arg residue generated by the cleavage acts as a degron for N-recognin ubiquitin ligase, leading to ubiquitination and proteasome-dependent degradation. Mutation of the ubiquitin C-terminal gly to val (G76V) prevents cleavage, generating a substrate where a ubiquitin chain is attached to the ubiquitin moiety (UFD reporter). (b) PLpro selectively stabilizes the N-degron reporter Ub-R-GFP. HEK293T cells transiently cotransfected with the Ub-R-GFP or Ub^G76V^-GFP reporters and FLAG-ev/PLpro/PLpro^mut^. FLAG-ev transfected cells were incubated overnight with 100 nM epoxomicin as a control for ubiquitin-dependent proteasomal degradation. Representative western blots from one out of three independent experiments are shown. (c) Densitometric quantification of the GFP-specific bands. The data are displayed as mean ± SD GFP intensity in PLpro/PLpro^mut^ transfected and epoxomicin-treated cells relative to FLAG-ev after normalization to the GAPDH loading control. Significance was calculated by unpaired two-tailed Student’s t-tests.

**Figure 3.**
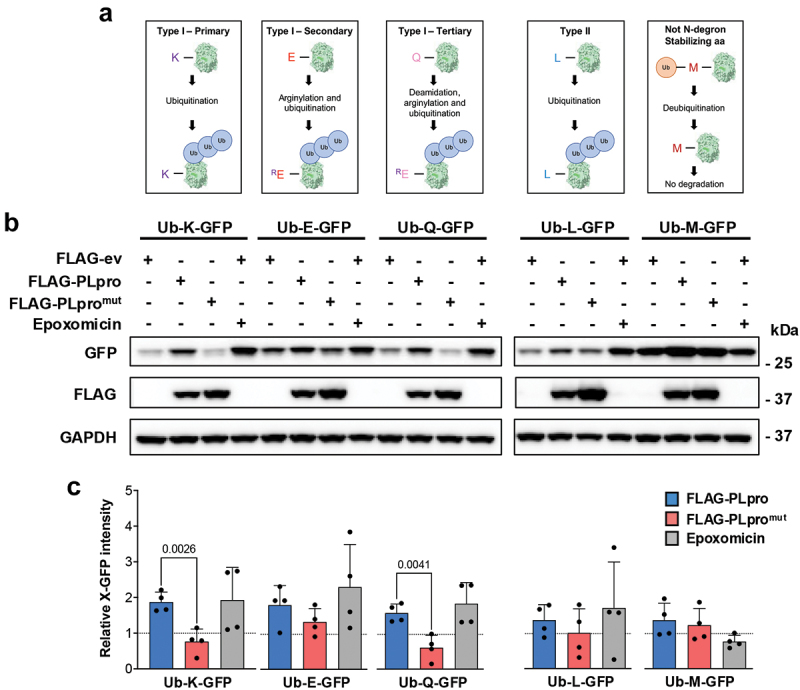
PLpro preferentially stabilizes type I N-degron substrates. (a) schematic illustration of the Ub-X-GFP reporters carrying type I (primary, secondary, and tertiary) or type II N-degrons and the control, Ub-M-GFP chimera that, upon ubiquitin cleavage, does not contain a degradation signal. (b) PLpro preferentially stabilizes type I N-degron substrates. HEK293T cells were cotransfected with the indicated Ub-X-GFP and FLAG-ev/PLpro/PLpro^mut^, and expression of the GFP chimeras was analyzed after 24 h by western blot. As a reference for ubiquitin-dependent proteolysis, one aliquot of FLAG-ev transfected cells was treated with 100 nM epoxomicin. Representative western blots from one out of four independent experiments are shown. (c) The intensity of the GFP band was quantified by densitometry in four independent experiments. The mean ± SD relative intensities of the GFP bands in FLAG-PLpro/PLpro^mut^ versus FLAG-ev transfected cells after normalization to the GAPDH loading control are shown. Significance was calculated by unpaired two-tailed Student’s t-tests.

N-degrons in mammals include type I (positively charged: R, K, and H) and type II (bulky hydrophobic: F, W, Y, L, and I) amino acids. In addition, the type I primary Nt-Arg degron can be generated by posttranslational modifications of the N-terminal amino acid via an enzymatic cascade, which involves arginylation (type I secondary residues: D, E) or deamination/oxidation followed by arginylation (type I tertiary residues: N, Q, C). To investigate whether PLpro may regulate the degradation of N-degron substrates, we first compared cells transfected with the active or inactive versions of the viral enzyme for the expression of previously described Ub-X-GFP fusion reporters containing either an Nt-Arg degron (Ub-R-GFP) or a C-terminal G to V mutation of the ubiquitin moiety (Ub^G76V^-GFP), which produces a ubiquitin fusion degradation (UFD) substrate where the Ub moiety serves as the site of ubiquitination by non-N-recognin ligases [54] (Figure 2A). In accordance with the notion that, upon hydrolysis of the Ub-bond by cellular deubiquitinases, the R-GFP reporter is efficiently ubiquitinated and degraded by the proteasome, a weak GFP band was detected in western blots of cells cotransfected with the FLAG-ev and the intensity of the band was strongly increased by treatment with the proteasome inhibitor epoxomicin (Figure 2B,C). A significant accumulation of R-GFP was also observed in cells expressing catalytically active FLAG-PLpro, whereas the catalytic mutant had no effect. Accumulation of the uncleaved Ub^G76V^-GFP reporter was observed only in cells treated with the proteasome inhibitor (Figure 2B,C), suggesting that PLpro may preferentially inhibit the degradation of N-degron-carrying substrates. To further explore this possibility, catalytically active and inactive PLpro were compared for their capacity to promote the accumulation of reporters carrying primary (Ub-K-GFP), secondary (Ub-E-GFP), or tertiary (Ub-Q-GFP) type I N-degrons or a type II N-degron (Ub-L-GFP) (Figure 3A). An Ub-M-GFP chimera that, upon release of the ubiquitin moiety, does not expose a degradation signal was used as a control. As expected, the expression of the M-GFP was not significantly affected by co-transfection of FLAG-PLpro/PLpro^mut^, whereas, consistent with the effect on R-GFP, catalytically active PLpro promoted the accumulation of K-GFP and Q-GFP at levels comparable to those achieved by inhibition of the proteasome (Figure 3B,C). A similar PLpro-dependent accumulation of E-GFP was observed although, due to experimental variation, the difference between catalytic active and mutant enzymes did not reach statistical significance. Of note, a small accumulation of the type II N-degron L-GFP was observed only occasionally in cells expressing catalytically active PLpro, suggesting that viral enzyme may preferentially stabilize substrates carrying type I N-degrons (Figure 3B,C). Cells expressing the membrane-anchored PLpro-TM exhibited an even stronger accumulation of the R-GFP reporter (Figure S3). Interestingly, although less pronounced, some stabilization of the reporter was also observed in cells expressing PLpro-TM^mut^, suggesting that overexpression of the large membrane-anchored polypeptide may affect the ubiquitin-proteasome system, possibly due to the presence of additional regulatory elements.

### PLpro stabilizes nt-arginylated ER chaperones

**Figure 4.**
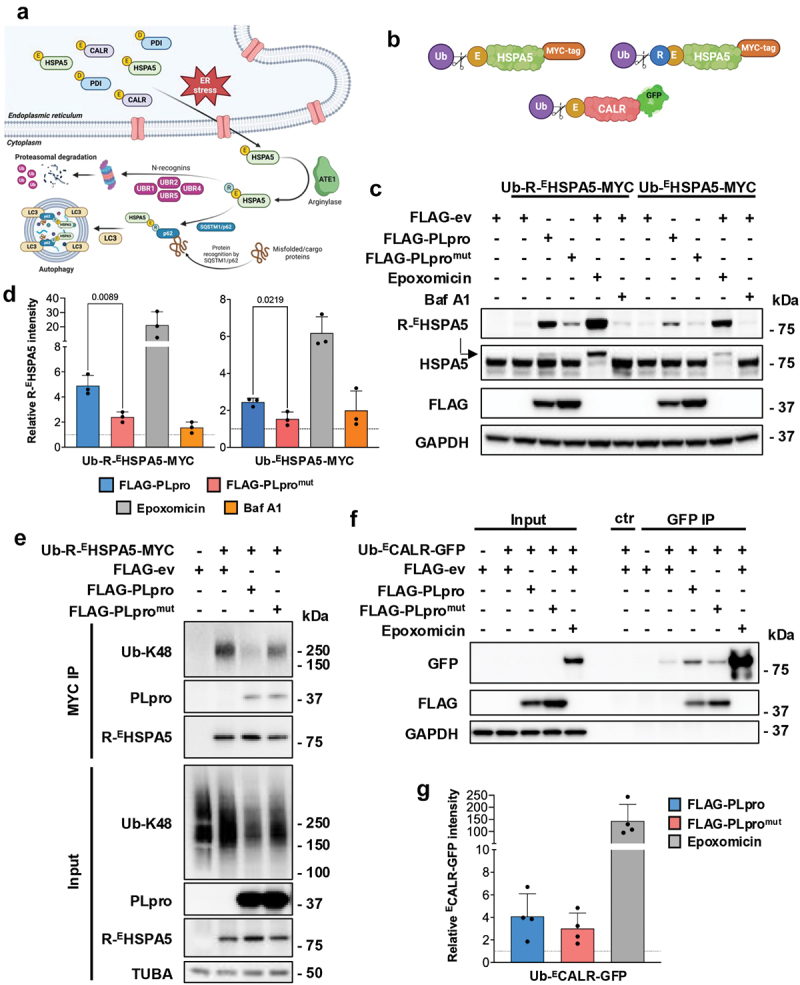
PLpro stabilizes N-degron-carrying ER chaperones. (a) Cartoon illustrating the generation of N-degron carrying ER chaperones upon induction of ER stress and involvement of N-degron carrying ^E^HSPA5 in the regulation of autophagy via activation of the N-recognin SQSTM1/p62. (b) Schematic illustration of the Ub-X-HSPA5-MYC and Ub-^E^CALR-GFP reporters. (c) PLpro promotes the stabilization of the ^E^HSPA5 and *R*-^E^HSPA5 reporters. HEK293T cells were transiently cotransfected with the FLAG-ev/PLpro/PLpro^mut^ and Ub-R^E^HSPA5-MYC or Ub-^E^HSPA5-MYC plasmids. As a control for proteasome- and lysosome-dependent degradation, the FLAG-ev transfected cells were treated overnight with 100 nM epoxomicin or 100 nM Baf A1, respectively. The expression of the *R*-^E^HSPA5 was analyzed 24 h post-transfection by probing western blots with the indicated antibodies. Representative blots from one out of three independent experiments are shown. (d) The intensities of the *R*-^E^HSPA5 bands were quantified by densitometry in three independent experiments. The mean ± SD relative intensity of the *R*-^E^HSPA5 bands in FLAG-PLpro/PLpro^mut^ transfected or epoxomicin/Baf A1 treated cells versus FLAG-ev transfected cells is shown. Significance was calculated by unpaired two-tailed Student t-test. (e) PLpro deubiquitinates *R*-^E^HSPA5. HEK293T cells were transiently cotransfected with Ub-R-^E^HSPA5-MYC and FLAG-ev/PLpro/PLpro^mut^. Cell lysates were immunoprecipitated with anti-MYC agarose beads, and western blots were probed with the indicated antibodies. Blots from one representative experiment out of two are shown in the figure. (f) PLpro stabilizes N-degron carrying CALR. HEK293T cells were cotransfected with FLAG-ev/PLpro/PLpro^mut^ and the Ub-^E^CALR-GFP. Cell lysates were immunoprecipitated with a GFP antibody, followed by capture with protein G-coated sepharose beads. An isotype-matched IgG control was included in the immunoprecipitation to verify specificity. Western blots were probed with the indicated antibodies. Blots from one representative experiment out of three are shown in the figure. (g) The intensities of the GFP bands were quantified by densitometry in three independent experiments. The mean ± SD, the relative intensity of the GFP bands in FLAG-PLpro/PLpro^mut^ versus FLAG-ev transfected cells is shown. Significance was calculated by unpaired two-tailed Student’s t-tests.

Natural N-degron substrates are generated by the processing of intracellular proteins by exo- or endopeptidase [55]. Well-documented examples include the pro-apoptotic Nt-Arg degron carrying species of BID (BH3 interacting domain death agonist), BCL2L1/BcL-xL (BCL2 like 1), and BCL2L11/BimEL (BCL2 like 11) that are generated by CAPN (calpain) or CASP3 (caspase 3) cleavage [56], and the signal peptidase processed ER chaperones HSPA5/BiP (heat shock protein family A (Hsp70) member 5), PDI (protein disulfide isomerase) and CALR (calreticulin) that expose secondary type I degrons (^E^HSPA5, ^D^PDI, ^E^CALR) and are arginylated upon translocation to the cytosol during ER stress [57]. *R*-^E^HSPA5 was shown to regulate autophagy via binding to the Nt-Arg degron-recognizing ZZ domain of SQSTM1/p62 [51,58] (Figure 4A). To test whether the interaction of PLpro with N-recognin ligases may lead to the stabilization of physiological substrates containing Nt-Arg degrons, we generated two Ub-X-HSPA5 fusion plasmids where X is either Glu, the endogenous secondary type I N-degron (Ub-^E^HSPA5) that is arginylated upon release of the Ub moiety, or R, the primary type I N-degron, which bypasses the need for arginylation of the reporter (Ub-R-^E^HSPA5, Figure 4B). The expression of *R*-^E^HSPA5 was monitored in western blots of HEK293T cells cotransfected with FLAG-ev/PLpro/PLpro^mut^ using a commercial *R*-^E^HSPA5-specific antibody. FLAG-ev cotransfected cells treated with epoxomicin or bafilomycin A_1_ (Baf A1) were included as controls for proteasome and lysosome-dependent degradation, respectively (Figure 4C). The transfected ^E^HSPA5 and *R*-^E^HSPA5 were hardly detected in western blots of cells cotransfected with the control FLAG-ev but were efficiently stabilized upon treatment with epoxomicin. In contrast, Baf A1 had no effect, confirming the behavior of the reporters as bone-fide N-degron proteasomal substrates (Figure 4C,D). Of note, lower levels of the ^E^HSPA5 reporter were regularly detected in epoxomicin-treated cells, suggesting that the expression levels of ATE1 may be rate-limiting for the generation of the proteasomal substrate. Both the *R*-^E^HSPA5 and the ^E^HSPA5 constructs were stabilized in cells expressing catalytically active PLpro, confirming the capacity of the viral enzyme to rescue physiologic N-degron substrates from proteasomal degradation (Figure 4C,D). In line with the known specificity of PLpro for K48-linked ubiquitin chains [11,25], significantly decreased levels of *R*-^E^HSPA5-MYC ubiquitination were observed when MYC-immunoprecipitates from lysates of cells expressing catalytically active PLpro were probed with a Ub-K48 specific antibody (Figure 4E). The effect appeared to be restricted to the N-degron substrate because the expression of PLpro had no appreciable effect on the total amounts of ubiquitin conjugates in the transfected cells (Figure S4A and S4B). The transfected ^E^HSPA5-MYC polypeptide was also stabilized in cells expressing the membrane-anchored PLpro-TM construct at levels comparable to those achieved by inhibition of the proteasome (Figure S5), supporting the conclusion that the native viral enzyme can efficiently counteract the degradation of the N-degron substrate.

To further assess whether PLpro may stabilize other N-degron-carrying substrates, the cells were co-transfected with PLpro or PLpro^mut^ and a ubiquitin-fusion chimera of a cytosol relocated version of the ER chaperone CALR (Ub-^E^CALR-GFP) (Figure 4B). ^E^CALR-GFP was only detected in the lysates of cells treated with epoxomicin, suggesting efficient arginylation and degradation by the proteasome (Figure 4F). Nevertheless, low-level accumulation of the reporter was reproducibly observed in PLpro-expressing cells when the product was enriched by GFP-tag immunoprecipitation (Figure 4F,G). Of note, PLpro was also detected in the GFP immunoprecipitates, confirming that the viral enzyme is recruited to and acts upon protein complexes containing the N-degron substrate.

### PLpro enhances the R-^E^HSPA5-driven oligomerization of SQSTM1/p62

**Figure 5.**
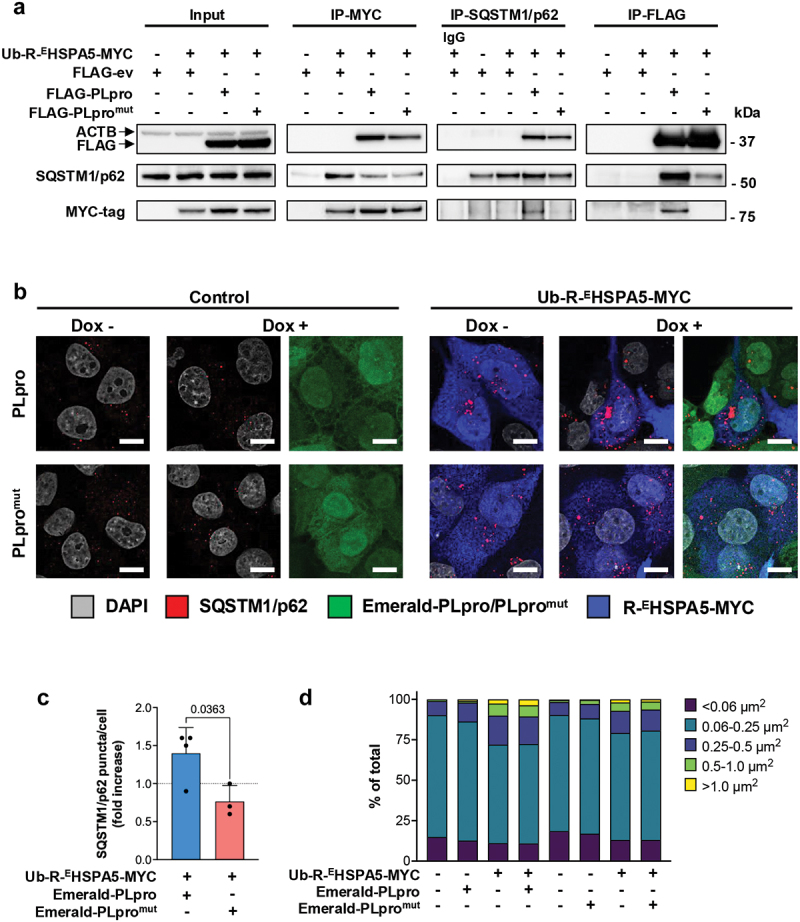
PLpro enhances the *R*-^E^HSPA5-driven formation of SQSTM1/p62 aggregates. (a) Reciprocal immunoprecipitation illustrating the interaction of PLpro with *R*-^E^HSPA5 and SQSTM1/p62. Equal aliquots of HEK293T lysates cotransfected with FLAG-ev/PLpro/PLpro^mut^ and Ub-R-^E^HSPA5-MYC were immunoprecipitated with anti-FLAG, anti-MYC or anti-SQSTM1/p62 antibody-coated beads. An isotype-matched antibody was used as a control. Western blots were probed with the indicated antibodies. Blots from one representative experiment out of three are shown in the figure. (b) Catalytically active PLpro enhances the formation of large SQSTM1/p62 aggregates in cells expressing *R*-^E^HSPA5. Endogenous SQSTM1/p62 (red) was detected by immunofluorescence in control and Dox-treated U2OS Emerald PLpro/PLpro^mut^ cells and transfected with the *R*-^E^HSPA5-MYC reporter (blue). Images from one representative experiment out of four are shown. (c, d) PLpro enhances the number and size of SQSTM1/p62 aggregates in *R*-^E^HSPA5-expressing cells. The number and size of SQSTM1/p62 puncta were determined using the Fiji software and its analysis particle function from 75 confocal images containing approximately 1000 cells per condition. For each image, the brightness and contrast were adjusted to reduce the background noise, and particles of size >0.049 μm^2^ were scored as puncta. (c) The data are presented as fold change in cells expressing *R*-^E^HSPA5 and PLpro/PLpro^mut^ relative to cells expressing *R*-^E^HSPA5 alone in three (PLpro^mut^) or four (PLpro) independent experiments. Significance was calculated by unpaired two-tailed Student’s t-tests. (d) Microsoft Excell was used to categorize the particles based on size. The data are represented as a percentage (%) of the total number of puncta. Data from the experiment in Figure 5B are shown. Similar results were obtained in three independent experiments. Scale bar: 10 µm.

It was previously shown that cytosolic *R*-^E^HSPA5 binds to the autophagy adaptor SQSTM1/p62, which triggers SQSTM1/p62 oligomerization, promotes the interaction with LC3, and activates macroautophagy [51,57]. To test whether PLpro may regulate this process, we first investigated whether PLpro is recruited to *R*-^E^HSPA5- and SQSTM1/p62-containing complexes. Cross immunoprecipitations were performed from cell lysates of HEK293T cells transfected with plasmids expressing Ub-R^E^HSPA5-MYC and FLAG-ev/PLpro/PLpro^mut^ using anti-FLAG or anti-MYC coated agarose beads or a SQSTM1/p62 specific antibody followed by capture of the immunocomplexes by protein G-coupled Sepharose beads (Figure 5A). In line with the stabilizing effect of the active enzyme, slightly higher levels of *R*-^E^HSPA5-MYC were detected in the lysates of FLAG-PLpro-expressing cells (Figure 5A, left panel). Both, catalytically active and inactive PLpro were readily detected in the *R*-^E^HSPA5-MYC and SQSTM1/p62 immunoprecipitates, and, as expected, SQSTM1/p62 was detected in the *R*-^E^HSPA5-MYC immunoprecipitates, independently of the presence of catalytically active or inactive PLpro. In contrast, *R*-^E^HSPA5-MYC was enriched in the SQSTM1/p62 immunoprecipitates of cells expressing catalytically active PLpro compared with the catalytic mutant. Only the active enzyme co-precipitated both SQSTM1/p62 and *R*-^E^HSPA5-MYC, pointing to an important role of the enzymatic activity in strengthening the trimolecular interaction.

To investigate the possibility, we then tested whether PLpro affects the formation of SQSTM1/p62 aggregates in *R*-^E^HSPA5-expressing cells. To avoid artifacts of overexpression, close to physiological levels of the viral enzyme were induced in U2OS cells stably transduced with recombinant retroviruses expressing Emerald-tagged PLpro/PLpro^mut^ under the control of a tetracycline-regulated promoter (Figure S6). U2OS cells were chosen for these experiments because more suitable for immunofluorescence studies. Of note, although clearly detected in all induced cells (Figure S6A), PLpro^mut^ was expressed at significantly lower levels than the catalytically active enzyme (Figure S6B). Based on the stabilizing effect of epoxomicin treatment, the lower steady-state levels are likely to be due to rapid turnover mediated by proteasomal degradation (Figure S6C). The presence of SQSTM1/p62 puncta was then monitored in control and induced cells with or without transfection of Ub-R-^E^HSPA5-MYC. Few SQSTM1/p62 aggregates were detected in the untreated cells by staining with a SQSTM1/p62-specific antibody, and the presence of the aggregates was only marginally affected by the induction of PLpro/PLpro^mut^ (Figure 5B, left panels). As expected, the number and size of the aggregates were significantly increased in cells expressing *R*-^E^HSPA5. A further increase in both aggregate number and size was observed in cells co-expressing catalytically active PLpro, while the catalytic mutant had no appreciable effect (Figure 5B right panels and Figure 5C), supporting the conclusion that the stabilization of *R*-^E^HSPA5 by viral deubiquitinase potentiates the induction of SQSTM1/p62 aggregates. Quantification of the size of the aggregates confirmed a relative increase in the proportion of aggregates of size >0.5 µm in cells expressing the active enzyme (Figure 5D).

### Components of autophagosome biogenesis machinery are recruited to PLpro-containing complexes

**Figure 6.**
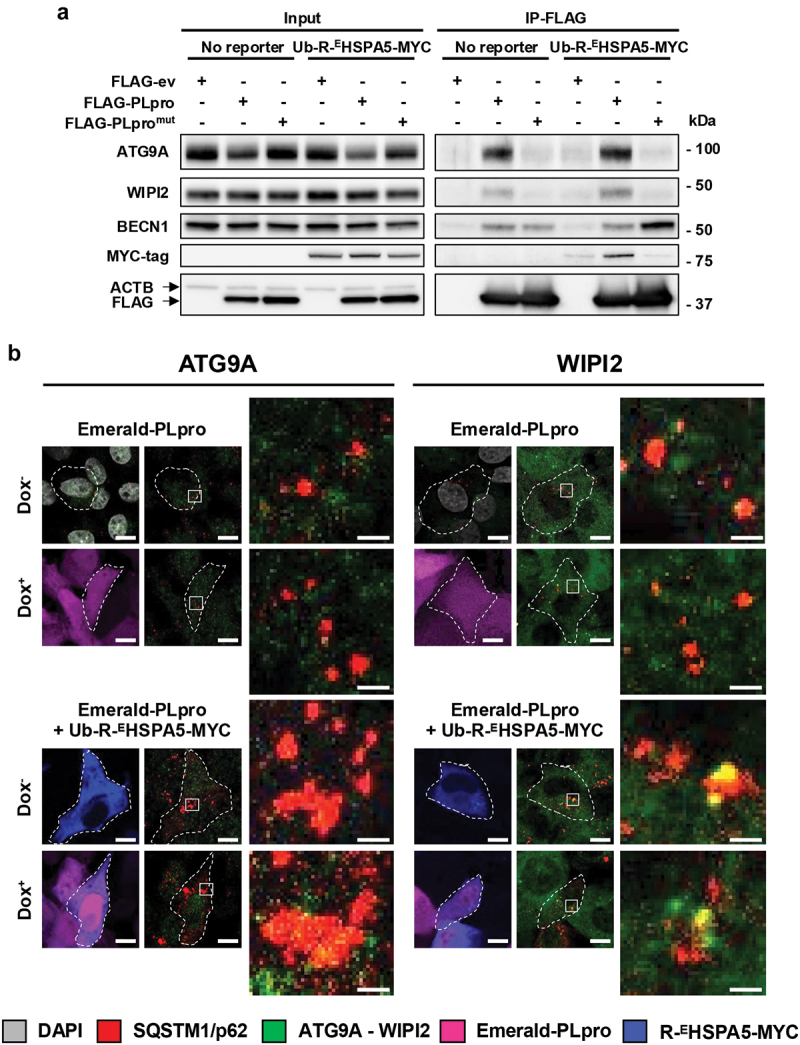
PLpro recruits components of the autophagosome biogenesis machinery to large SQSTM1/p62 aggregates. (a) FLAG immunoprecipitates of HEK293T cells transfected with FLAG-ev/PLpro/PLpro^mut^ in the presence or absence of co-transfected Ub-R-^E^HSPA5-MYC were probed with the indicated antibodies. Western blots from one representative experiment out of three are shown in the figure. (b) Representative confocal images illustrating the colocalization of ATG9A and WIPI2 with large SQSTM1/p62 aggregates. Scale bar: 10 µm.

Recent evidence suggests that large SQSTM1/p62 bodies gather components of the ULK1 (unc-51 like autophagy activating kinase 1) and ULK2 and class III phosphatidylinositol 3-kinase (PtdIns3K) complexes [59–61] as well as ATG9A and ATG16L1-decorated vesicles [61], serving as nucleation sites for autophagosome biogenesis. To test whether PLpro may affect this process, we tested whether it interacts with components of the autophagy machinery. FLAG immunoprecipitates of HEK293T cells transfected with FLAG-ev/PLpro/PLpro^mut^ in the absence or presence of Ub-R-^E^HSPA5-MYC were probed with a selection of antibodies specific for ATG9A, the catalytic subunit of the ULK1-ATG13-ATG101-RB1CC1/FIP200 kinase complex ULK1, the PIK3C3/VPS34 and BECN1 components of the PtdIns3K complex 1, the WIPI2 (WD repeat domain, phosphoinositide interacting 2) and WDR45/WIPI4 (WD repeat domain 45) subunits, the lipid-transfer protein ATG2A, the ATG12–ATG5 and ATG16L1 components of the ATG12–ATG5-ATG16L1 ubiquitin-like conjugation system, and LC3. ATG9A and WIPI2 were readily detected in the immunoprecipitates of catalytically active PLpro alongside *R*-^E^HSPA5, whereas only BECN1 was detected in the immunoprecipitates of both the active and inactive enzyme (Figure 6A). Immunofluorescence staining confirmed the colocalization of ATG9A and WIPI2 with the large SQSTM1/p62 aggregated detected in cells expressing *R*-^E^HSPA5, and the interaction with ATG9A vesicles appeared to be enhanced in the presence of catalytically active PLpro (Figure 6B).

**Figure 7.**
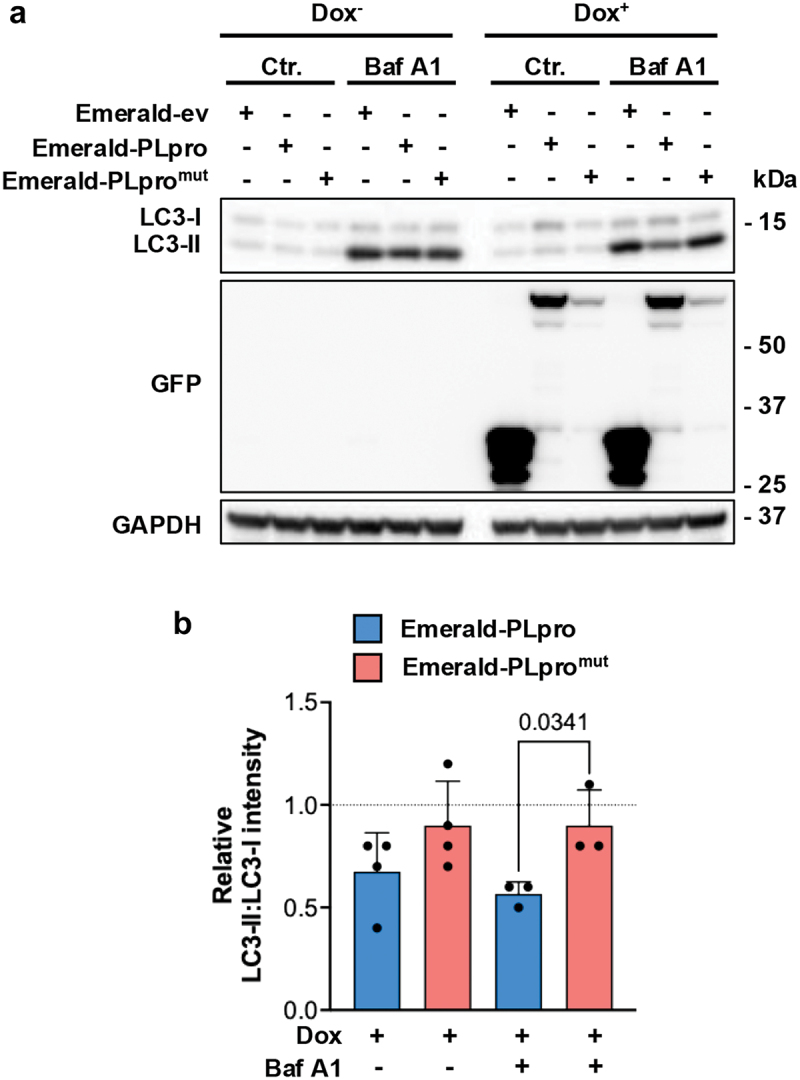
Catalytically active PLpro inhibits the formation of LC3-II. (a) Control and doxycycline-treated U2OS cells stably transfected with plasmids expressing inducible Emerald-ev/PLpro/PLpro^mut^ were kept untreated or treated overnight with 100 nM Baf A1 before analysis of protein expression by probing western blots with the indicated antibodies. Blots from one representative experiment out of four are shown. (b) Quantification of the LC3-specific bands. The data are presented as LC3-II:LC3-I ratios in four independent experiments. Significance was calculated by unpaired two-tailed Student’s t-tests.

Other components of the autophagy machinery were not found in the immunoprecipitates (Figure S7). These included ULK1 that, in line with the previous finding that PLpro cleaves ULK1 at an LGGG consensus sequence [62], was hardly detected in cells expressing the catalytically active enzyme (Figure S7A). The effect of PLpro on ULK1 expression was further confirmed upon induction of PLpro/PLpro^mut^ in the U2OS transduced cell lines (Figure S7B and S7C). ULK2, PIK3C3/VPS34, WDR45/WIPI4, ATG2A, components of the ATG12–ATG5-ATG16L1 lipid-conjugation system, and LC3 were also absent (Figure S7A). The failure to recruit both LC3 and the ATG12–ATG5-ATG16L1 ligase suggests that the production of lipidated LC3 may be impaired in PLpro-expressing cells. To test this possibility controls and Dox-treated U2OS-Emerald-ev/PLpro/PLpro^mut^ cells were cultured overnight in the absence or presence of 100 nM Baf A1 (Figure 7A,B). The expression of the active enzyme correlated with a significantly decreased accumulation of LC3-II in Baf A1 treated cells, which is consistent with impaired conversion of LC3-I to the lipidated LC3-II species.

### PLpro inhibits reticulophagy

**Figure 8.**
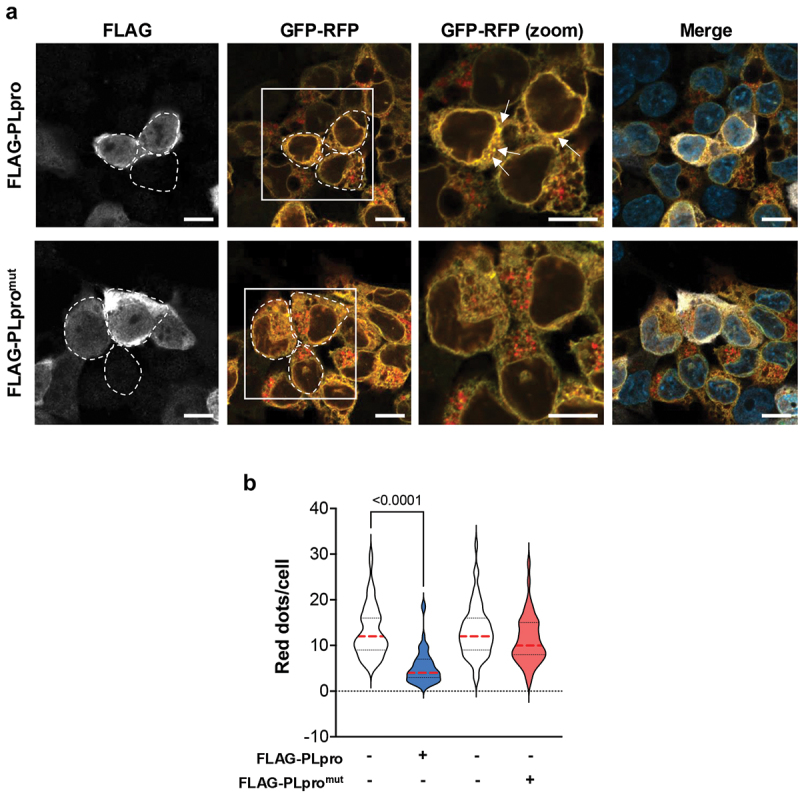
PLpro inhibits reticulophagy. (a) Representative confocal images illustrating the failure to accumulate ER-loaded autophagolysosomes in cells expressing active PLpro. The EATR reporter expresses in-frame the coding sequence of the SERP1/RAMP4 subunit of the ER translocon complex followed by the coding sequences of eGFP and ChFP. Upon ER insertion of the SERP1 domain, eGFP and ChFP face the cytosol and emit equal fluorescence, whereas, due to the selective loss of eGFP fluorescence at low pH, er-loaded autophagosomes appear as distinct red fluorescent dots. Stable HCT116-EATR cells were transfected with plasmids expressing FLAG-PLpro/PLpro^mut^ and then starved overnight in EBSS medium before visualizing the formation of er-loaded autophagosomes by confocal microscopy. Yellow dots corresponding to ER membrane aggregates in cells expressing catalytically active PLpro are indicated by arrows. Scale bar: 10 µm. (b) Quantification of the number of red fluorescent dots in FLAG-PLpro/PLpro^mut^ positive and negative cells from the same transfection experiments. The cumulative data from two independent experiments where ≥50 PLpro positive and ≥50 negative cells were scored are shown. Significance was calculated by unpaired two-tailed Student t-tests.

The oligomerization of SQSTM1/p62 induced by the binding of Nt-Arg substrates to the ZZ domain was shown to trigger reticulophagy [63]. To test whether PLpro impacts reticulophagy, we made use of a previously described subline of HCT116 expressing a Dox-regulated reticulophagy tandem fluorescence reporter (HCT116-EATR) constructed by in-frame fusion of the coding sequences of the SERP1/RAMP4 subunit of the ER translocon, enhanced GFP (eGFP), and mCherry Fluorescent Protein (ChFP) [64]. Upon ER insertion of the SERP1 domain, GFP and ChFP face the cytosol and emit equal fluorescence, whereas, due to the selective loss of GFP fluorescence at low pH, ER-loaded phagolysosomes appear as distinct red fluorescent dots. The reporter cell line was transfected with plasmids expressing FLAG-PLpro/PLpro^mut^ and cultured for 24 h in the presence of Dox before being starved overnight in EBSS medium, followed by visualization of phagolysosomes by confocal microscopy. Red cargo-loaded phagolysosomes were readily detected in cells expressing the mutant PLpro at levels comparable to those observed in the non-transfected cells in the same sample. In contrast, diffuse yellow fluorescence and distinct yellow dots, presumably corresponding to aggregates of the ER reporter engulfed in autophagosomes, were observed in most cells expressing the active PLpro (Figure 8A). Quantification of the number of red dots in PLpro/PLpro^mut^-positive and -negative cells from the same transfection revealed a highly significantly impaired fusion of lysosomes with ER-loaded autophagosomes in cells expressing the catalytically active PLpro (Figure 8B), supporting the conclusion that the viral enzyme inhibits a late step in the reticulophagy pathway.

## Discussion

Similar to the PLpro encoded by other coronaviruses, the SARS-CoV-2 PLpro recognizes both ISG15 and ubiquitin conjugates, but due to the strong preference for ISG15 chains detected in various *in vitro* and cellular assays, the putative substrates of the deubiquitinase activity have received less attention. By focusing our analysis on the ubiquitin ligases that interact with the isolated PLpro domain, we found that the vDUB may participate in various protein complexes containing cellular E3s ligases involved in the regulation of antiviral responses. The interaction with several members of the N-recognin ligase family appeared particularly interesting in view of the reported upregulation of ATE1 in SARS-CoV-2 infected cells [53] and the prominent changes in the abundance and pattern of protein arginylation detected in the plasma of COVID-19 patients [65]. By testing the effect of PLpro on a set of reporter substrates that distinguish between N-degron-dependent versus other types of ubiquitination-induced proteasomal degradation, we observed selective stabilization of the N-degron reporters, with a preference for type I degrons where basic N-terminal residues, such as Arg, are detected by the N-recognin ligases [50]. Most importantly, we found that PLpro stabilized N-degron-carrying versions of the ER-chaperones HSPA5/BiP and CALR that become physiological substrates of the ATE1 arginyl-transferase upon release into the cytosol during ER stress [57], and selectively removed K48-linked polyubiquitin chains from R-HSPA5. It is noteworthy that endogenous *R*-^E^HSPA5 did not accumulate in cells expressing the PLpro catalytic domain alone. Thus, it appears that the viral enzyme may not be directly involved in the induction of ER stress but could play an important role in modulating the cellular response to ER stress during infection.

Many ER-targeted proteins acquire the potential to undergo N-terminal arginylation upon signal peptide cleavage and retrotranslocation to the cytosol [57], highlighting an important role of the N-degron pathway in the turnover and function of ER-resident proteins. ER chaperones that relocate to other cellular compartments under ER stress regulate various cellular functions, including transcription [66], mitochondrial homeostasis [67], and apoptosis [68]. Upon transport to the cell surface, ER chaperones were shown to modulate signaling pathways, including TGFB signaling [69], promote PtdIns3P production via association with PtdIns3K [70], and serve as coreceptors for virus internalization [70,71], with HSPA5 being upregulated and exerting pro-viral functions during SARS-CoV-2 infection [72,73]. N-terminally arginylated HSPA5 was shown to regulate autophagy through binding of the Nt-Arg residues to the ZZ domain of the N-recognin SQSTM1/p62, which promotes SQSTM1/p62 self-polymerization, recruits lipidated LC3-II, and targets the chaperone, presumably together with client proteins, to autophagy [57,74,75]. We found that catalytically active PLpro binds with both *R*-^E^HSPA5 and endogenous SQSTM1/p62 in reciprocal co-immunoprecipitation assays, and the active enzyme promoted a significant increase in the formation of SQSTM1/p62 aggregates induced by *R*-^E^HSPA5. Even though we have not formally proven that the formation of SQSTM1/p62 aggregates is *R*-^E^HSPA5-dependent under our assay conditions, as opposed to possible artifacts of protein overexpression, the capacity of PLpro to interact with and counteract the activity of N-recognin ligases rescuing *R*-^E^HSPA5 from proteasomal degradation, together with the selective binding of catalytically active PLpro to SQSTM1/p62 and *R*-^E^HSPA5 and its enhancing effect on both number and size of *R*-^E^HSPA5-induced SQSTM1/p62 aggregates, is consistent with a key role of the PLpro-mediated stabilization of *R*-^E^HSPA5 in aggregate formation.

Although the capacity of SQSTM1/p62 aggregates to promote autophagosome biogenesis is well established, their precise role in the process is not fully understood. While SQSTM1/p62 can tether LC3-decorated isolation membrane through direct SQSTM1/p62-LC3 interaction [76,77], SQSTM1/p62 also interacts with RB1CC1 and may thus initiate autophagosome formation via recruitment of the ULK1 complex [59,60]. In line with the latter scenario, combined biochemical reconstitution and cellular assays have recently confirmed the capacity of large SQSTM1/p62 aggregates to gather ATG9A positive vesicles and provide a platform for ULK1 complex assembly, PtdIns3K activation, and the generation of PtdIns3P [61,78]. It is noteworthy that the assembly of ATG9A vesicle and recruitment of the core autophagosome machinery occurred independently of binding to LC3, suggesting that the formation of SQSTM1/p62 aggregates is sufficient to initiate phagophore assembly. This conclusion is further substantiated by the finding that the knockdown of all members of the LC3/GABARAP family, while indispensable for autophagosome-lysosome fusion, did not prevent the formation of sealed autophagosomes [79]. The interaction of PLpro with both R-HSPA5 and SQSTM1/p62, together with the formation of large SQSTM1/p62 aggregates in cells expressing that active enzyme, suggests that PLpro may regulate the aggregate-driven biogenesis of autophagosome. In line with this possibility, we found that PLpro interacts with the PtdIns3K regulatory subunit BECN1, while only the active enzyme interacts with ATG9A and with WIPI2 that is recruited to the phagophore membrane by binding to PtdIns3P [80]. WIPI2 and ATG9A also colocalized with the large SQSTM1/p62 aggregates induced in the presence of *R*-^E^HSPA5, and PLpro appeared to enhance the recruitment of ATG9A-positive vesicles that serve as a seed for autophagosome biogenesis.

In sharp contrast to the presence of PLpro in protein complexes that drive the early steps of autophagosome biogenesis, we found that PLpro does not interact with the ATG12–ATG5-ATG16L1 ligase complex that is brought to the site by binding to WIPI2 [81], nor with LC3. We also confirmed previous reports demonstrating that PLpro catalyzes the hydrolysis of the peptide bonds C-terminal to an LGGG motif in ULK1, which separates the kinase domain from the substrate recognition domain [62,82]. ULK1 was shown to play a crucial role in the early steps of autophagosome biogenesis through the phosphorylation of members of the PIK3C3/VPS34 kinase complex and their binding partners [83]. However, while inhibiting ULK1 activity by small molecules blocks autophagy, it does not prevent the recruitment of the PtdIns3P-forming complex to autophagosome-like structures that are, however, abnormal in size and do not traffic to the lysosome [84]. Our finding that PLpro inactivates ULK1 has two important implications. First, it infers that other kinases are likely involved in PtdIns3K activation. These may include the ULK1 homolog ULK2, which exhibits important differences in autophagy-related interactions, post-transcription, and transcriptional regulation [85], and is not a substrate of the PLpro protease. In addition, it points to an important role of ULK1 in regulating the late stages of autophagy. Indeed, both ATG16L1 [86] and its binding partner WIPI2 [87] were shown to serve as ULK1 substrates, which, depending on the triggering, may either activate or inhibit autophagy.

The N-degron-induced oligomerization of SQSTM1/p62 was shown to trigger reticulophagy via binding to the ER membrane ubiquitin ligase TRIM13 that is auto-ubiquitinated via Lys63 linkage, which facilitates the recruitment of LC3 to the site of phagophore formation [3,63]. Our finding that LC3 is not found in the PLpro immunoprecipitates suggests that the recruitment of the viral protein to SQSTM1/p62 aggregates could halt this process. Since PLpro does not hydrolyze K63-linked ubiquitin chains, it seems unlikely that the deubiquitinase would affect the interaction of SQSTM1/p62 with ubiquitinated TRIM13. Thus, the inhibition of reticulophagy may be directly linked to the failure to generate LC3-II. Indeed, we found that catalytically active PLpro inhibits the acidification of ER-loaded autophagosomes, while puncta corresponding to reporter aggregates were often observed in cells expressing the active enzyme, suggesting that the formation of autophagic vesicles is not affected. This finding provides an interesting addition to the complex interaction of SARS-CoV-2 with autophagy, where several viral proteins, including ORF3a [88], ORF7a [89], ORF10 [90], NSP6 [91], and NSP13 [92] were shown to alter the activity of multiple components of the pathways, resulting in both activating and inhibitory effects [93,94]. Like many other RNA viruses, SARS-CoV-2 exploits the cellular autophagy machinery to remodel the cellular membranes toward the production of double-membrane vesicles (DMV) that provide a suitable environment where virus replication is concealed from the host antiviral response [95]. The ATG12–ATG5-ATG16L1 complex was shown to be dispensable for SARS-CoV-2 replication, whereas other components of the autophagosome biogenesis machinery, such as elements of the PtdIns3K complex, are critically involved, most likely by contributing to the formation of DMVs [96]. Together with Nsp4 and Nsp6, Nsp3 was shown to play a key role in DMVs biogenesis, with the protease function of the PLpro domain being essential for cleavage of the Nsp3-Nsp4 bond and for the formation of transmembrane pores [97] and ordered lamellar structures [4]. Our findings suggest a possible additional role of the deubiquitinase activity of PLpro in triggering the activation of SQSTM1/p62 and selective recruitment of components of the autophagy machinery, which may promote the expansion of DMV membranes while inhibiting their destruction by reticulophagy.

## Materials and methods

### Reagents

For a complete list of reagents, kits, and commercially available or donated plasmids with source identifiers, see Table 1. For a list of primers used for cloning and PCR analysis, see Table 2.

**Table 1.** Reagents used in the investigation.

Reagent	Source	Catalog number
**Chemicals, peptides, and recombinant proteins**		
IGEPAL CA-630	Sigma-Aldrich	I3021
Polyvinylpyrrolidone 40kDa (PVP-40)	Sigma-Aldrich	PVP40
Nonfat dried milk powder	PanReac AppliChem	A0830
Tris-HCl pH 7.4 (1 M)	AccuGENE^TM^	51237
Sodium chloride	Fisher Scientific	S/3160/60
Calcium chloride	Sigma-Aldrich	C-2661
Magnesium chloride (1 M)	Invitrogen	AM9530G
Sodium dodecyl sulphate	Sigma-Aldrich	L3771
N-Ethylmaleimide	Sigma-Aldrich	E1271
Iodoacetamide	Sigma-Aldrich	I1149
EDTA pH 8.0 (0.5 M)	Invitrogen	AM9260G
Sucrose	Duchefa biochemie	S0809
Dithiothreitol (DTT)	Sigma-Aldrich	D9779
Triton X-100	Sigma-Aldrich	T9284
Bovine serum albumin	Sigma-Aldrich	A7906
Agarose	Sigma-Aldrich	A9539
Tween-20	Sigma-Aldrich	P9416
Glycerol solution	Sigma-Aldrich	49781
Trizma base	Sigma-Aldrich	93349
Glycine	Fisher Scientific	G/0800/60
Methanol	Fisher Scientific	M/4000/PC17
Ciprofloxacin	Sigma-Aldrich	17850
Doxycycline cyclate	Sigma-Aldrich	D9891
Epoxomicin	Millipore	324801
Cycloheximide	Sigma-Aldrich	C4859
Bafilomycin A_1_	Thermo Scientific	J67193.XF
Polybrene	Sigma-Aldrich	TR-1003-G
Complete protease inhibitors cocktail	Roche Diagnostic	04693116001
Phosphatase inhibitor cocktail	Roche Diagnostic	04906837001
DAPI	Sigma-Aldrich	9542
Mowiol	Calbiochem	475904
HA-Ubiquitin-Vinyl sulfone (VS)	R&D Systems	U-212-025
ISG15-VS	BostonBiochem	UL-603
Ac-hISG15prox-VPS Vinyl pentynyl sulfone (VPS)	UbiQ	B01065021–001 (UbiQ-311)
**Kits**		
DC Protein Assay quantification kit	Bio-Rad	A500–0116
Albumin standard solution	Thermo Scientific	23210
SuperSignal^TM^ West Pico PLUS Chemiluminescent Substrate	Thermo Scientific	XE356732
SuperSignal^TM^ West Femto Maximun Sensitivity Chemiluminescent Substrate	Thermo Scientific	34096
jetOPTIMUS DNA transfection reagent	Polyplus	101000006
Q5® Site-Directed Mutagenesis Kit	New England Biolabs	E0554S
QIAprep® Spin Miniprep Kit	QIAGEN	27106
ZymoPURETM II Plasmid Maxiprep Kit	Zymo Research	D4203-A
Monarch® DNA Gel Extraction Kit	New England Biolabs	T1020S
QIAquick® PCR Purification Kit	QIAGEN	28104
**Experimental models: Cell lines**		
HeLa	ATCC	RR-B51S
HEK293T	ATCC	CRL3216
U2OS	ATCC	HTB-96
HCT116	ATCC	
HECT116-EATR	Liu et al. [26]	
**Cell culture media**		
DMEM	Sigma-Aldrich	D6429
Gibco™ Earle’s Balanced Salt Solution (EBSS)	Fisher Scientific	24010–043
**Recombinant DNA**		
p3XFLAG-CMV™-10	Sigma-Aldrich	E7658
PLproCoV2_pCMV-3Tag-1a	This work	GeneScript
PLproCoV2-C111A_pCMV-3Tag-1a	This work	GeneScript
NSP3-mCherry	Addgene	165131; Bruno Antonny lab
mEmerald-C1	Addgene	53975; Michael Davidson lab
mEmerald-PLpro	This work	N/A
mEmerald-PLpro^C111A^	This work	N/A
FLAG-Empty vector (based on mEmerald-C1)	This work	N/A
FLAG-PLpro	This work	N/A
FLAG-PLpro^C111S^	This work	N/A
FLAG-PLpro-TM	This work	N/A
FLAG-PLpro-TM^C111S^	This work	N/A
Ub-R-GFP	Dantuma at al [27].	N/A
Ub-G76V-GFP	Dantuma et al. [27]	N/A
Ub-M-GFP	Dantuma et al. [27]	N/A
Ub-K-GFP	This work	N/A
Ub-E-GFP	This work	N/A
Ub-Q-GFP	This work	N/A
Ub-L-GFP	This work	N/A
pCMV BiP-MYC-KDEL-wt	Addgene	27164; Ron Prywes lab
pTRIPZ	Horizon Discovery	Inducible Dharmacon™ TRIPZ™ Lentiviral shRNA
pMD2.G	Addgene	12259; Didier Trono lab
psPAX2	Addgene	12260; Didier Trono lab
pTRIPZ- mEmerald-PLpro/PLpro^C111A^	This work	N/A
pEGFP N1 Ub-CRT	Addgene	69619; Marta Hallak lab

**Table 2.** PCR primers^a.^.

Gene	Forward primer (5’-3’)	Reverse primer (5’-3’)
mEmerald-PLpro/PLpro^C111A^ (for insertion in pTRIPZ vector)	TTTTTTAAGCTTtgGAAGTGAGAACCATCAAGGTC	TTTTTTGAATTCTTATTTGATTGTTGTAGTGTATGAGTTTTC
mEmerald-PLpro-TM (for amplification PLpro-TM to be inserted in emerald vector)	TTTTTTCCGGAGTGAGGACCATCAAGGTGT	TTTTTAGATCTttaCTTCAGGGCGATCTTGGT
FLAG-PLpro-TM (to change emerald tag to flag)	gatgatgataaagattataaagatgatgatgataaaTCCGGAGTGAGGACC	atctttataatctttatcatcatcatctttataatcGACCGGTAGCGCTAG
FLAG-PLpro (to remove TM)	TAAAGATCTCGAGCTCAAG	CTTGATGGTGGTGGTGTAG
FLAG-Empty vector (remove PLpro-TM)	AGATCTCGAGCTCAAG	TCCGGATTTATCGTCATC
FLAG-PLpro^C111S^ and FLAG-PLpro-TM^C111S^	CGACAACAACtctTACCTGGCCAC	GCCCACTTGATGCTGGTC
Ub-K-GFP (Site directed mutagenesis)	CAGAGGTGGGaagGGGAAGCTTGGTC	AGACGGAGTACCAGGTGC
Ub-E-GFP (Site directed mutagenesis)	CAGAGGTGGGgagGGGAAGCTTGGTC	AGACGGAGTACCAGGTGC
Ub-Q-GFP (Site directed mutagenesis)	CAGAGGTGGGcagGGGAAGCTTG	AGACGGAGTACCAGGTGC
Ub-L-GFP (Site directed mutagenesis)	CAGAGGTGGGctgGGGAAGCTTG	AGACGGAGTACCAGGTGC
Ub-E-BiP-MYC	GGTGGTCGTCTCAGAGGTGGGGAGGAGGAG	GGTGGTGCGGCCGCCTACAGCTCGTCCTTC
Ub-R-^E^BiP-MYC (Site directed mutagenesis)	AGGTGGGcgcGAGGAGGAGGACAAGAAGGAGG	TCCTCgcgCCCACCTCTGAGACGGAG

a. Inserted sequences are in lower case, and restriction sites are underlined.

### Antibodies

Primary antibodies – Mouse monoclonal: anti-ACTB/β-actin clone AC-15 (Sigma-Aldrich, A5441; 1:5000); anti-GAPDH (Millipore, CB1001; 1:5000); anti-TUBA/tubulin (Millipore, CP06; 1:2000); anti-FLAG (Sigma-Aldrich, F3165; 1:10000); anti-GFP (B-2; Santa Cruz Biotechnology, sc-9996; 1:1000); anti-UBR1 (Clone 6H9 1; Millipore, MABS1180; 1:2000); anti-Ub (P4D1; Santa Cruz Biotechnology, sc-8017; 1:1000); anti-SQSTM1/p62 Ick ligand (BD Biosciences 610,832; WB: 1:1000; IF: 1:200); anti-BECN1 (Proteintech 66,665–1-Ig; 1:1000); anti-WIPI2 (Abcam, ab105459; WB: 1:1000); anti-MYC Tag (Proteintech 60,003–2-Ig; IF: 1:200); anti-SQSTM1/p62-CoraLite®594-conjugated (Proteintech, CL594–66184; IF: 1:150).

Rabbit monoclonal: anti-MYC Tag (Cell Signaling Technology, 2278; WB: 1:1000; IF: 1:200); anti-HSPA5/BiP (Cell Signaling Technology, 3177S; 1:1000); anti-Ub-K48 (clone Apu2; Millipore, 05–1307; 1:1000); anti-ATG9A (Abcam, ab108338; WB:1:1000); anti-ATG12 (Cell Signaling Technology, 4180S; 1:1000).

Rabbit polyclonal: anti-FLAG (Sigma-Aldrich, F7425; IF: 1:400); anti-PLpro SARS-CoV-2 (GeneTex, GTX135796; 1:2000); anti-UBA6 (Thermo Fisher Scientific, A304-106A; 1:1000); anti-UBR2 (Abcam, ab217069; 1:2000); anti-UBR4 (Abcam, ab86738; 1:2000); anti-UBR5 (anti-EDD; Abcam, ab70311; 1:2000); anti-HSPA5/BiP (Abcam, ab21685; 1:1000); anti-HSPA5/BiP, arginylated (N-Glu19; Millipore, ABS2103; 1:1000); anti-LC3B (Sigma-Aldrich, L7543; WB: 1:2000; IF: 1:200); anti-ULK1 (Proteintech 29,005–1-AP; 1:1000); anti-ULK2 (Thermo Fisher Scientific, PA5–22173; 1:1000); anti-PIK3C3/VPS34 (Proteintech 12,452–1-AP; 1:1000); anti-ATG9A (Proteintech 26,276–1-AP; IF: 1:200); anti-ATG2A (Proteintech 23,226-1AP; 1:1000); anti-ATG5 (Proteintech 10,181–2-AP; 1:1000); anti-ATG16L1 (Proteintech 29,445–1-AP; 1:1000); anti-WIPI2 (Proteintech 28,820–1-AP; IF: 1:200); anti-WDR45/WIPI4 (Proteintech 19,194–1-AP; 1:1000).

Secondary antibodies – Donkey Anti-Rabbit IgG (H+L) Antibody, Alexa Fluor 488 (Thermo Fisher Scientific, A-21206; 1:1000); Donkey anti-Mouse IgG (H+L) Antibody, Alexa Fluor 555 (Thermo Fisher Scientific, A-31570; 1:1000); Goat Anti-Rabbit IgG (H+L) Antibody, Alexa Fluor 647 (Thermo Fisher Scientific, A-21245; 1:1000); Goat Anti-Mouse IgG (H+L) Antibody, Alexa Fluor 750 (Thermo Fisher Scientific, A-21037; 1:1000).

Agarose and Sepharose beads – Anti-FLAG M2 affinity gel (Sigma-Aldrich, A2220); ChromoTek MYC-Trap agarose beads (Proteintech, yta); Agarose TUBE 1 (His6 SUMO; LifeSensors, UM401); Protein-G coupled Sepharose beads (GE Healthcare, 17-0885-01). Isotype controls – Rabbit IgG (Abcam, ab172730); Mouse IgG2b kappa (eBMG2b; Thermo Fisher Scientific, 14-4732-82).

### Plasmid construction

For all standard cloning, subcloning procedures, and site-directed mutagenesis, commercial NEB DH5-alpha chemically competent *E. coli* (New England Biolabs, C2987H) was used. Standard PCR for amplification was performed with the Phusion High-Fidelity DNA Polymerase (New England Biolabs, M0530S) according to the manufacturer’s instructions. The cDNAs coding for SARS-CoV-2 (MN908947.3) Nsp3 residues 746–1060 (FLAG-PLproCoV2_pCMV-3Tag-1a, FLAG-PLpro) and the catalytic mutant carrying a Cys111 to Ala substitution (FLAG-PLproCoV2^C111A^_pCMV-3Tag-1a, FLAG-PLpro^mut^) were synthesized by GenScript (https://www.genscript.com/) with 5’EcoRI and 3’ EcoRV sites. For the functional analysis, the SARS-CoV-2 Nsp3 sequence (MN908947.3) from the Nsp3-mCherry plasmid was used as a template to amplify the PLpro (residues 746–1060) and PLpro-TM (residues 746–1943) sequences using primers containing BspEI and BglII restriction sites and cloned into the BspEI and BglII sites of the mEmerald-C1 vector yielding the mEmerald-PLpro plasmid. Catalytic mutants carrying a Cys111 to Ser substitution were generated by site-directed mutagenesis using the Q5® Site-Directed Mutagenesis Kit according to the recommended protocol. The Emerald tag of the mEmerald-PLpro plasmid was replaced by the FLAG tag by site-directed mutagenesis. For lentivirus transduction, the mEmerald-PLpro/PLpro^mut^ coding sequence was amplified with primers containing the AgeI and MluI restriction sites, followed by ligation in the same sites of the TRIPZ Inducible Lentiviral shRNA (Horizon Discovery) vector. The N-degron reporter plasmids Ub-R-GFP and Ub-M-GFP and the UFD reporter plasmid Ub^G76V^-GFP were previously described [54]. Other Ub-X-GFP reporters (X= K, E, Q, and L) were produced by substituting the Arg codon in the Ub-R-GFP plasmid by site-directed mutagenesis. The Ub-^E^HSPA5-MYC plasmid was constructed by replacing the R-GFP coding sequence from the Ub-R-GFP plasmid with the ^E^HSPA5-MYC coding sequence extracted by PCR from the pCMV-HSPA5-MYC-KDEL-wt using primers containing the BsmBI and NotI restriction sites followed by cloning into the BsmBI and NotI sites of the Ub-R-GFP plasmid. To construct the Ub-R-^E^HSPA5-MYC plasmid, the Q5® Site-Directed Mutagenesis Kit was used to add an N-terminal Arg codon to the ^E^HSPA5 sequence. All constructs and sub-constructs were verified by restriction digest and by Sanger Sequencing.

### Lentivirus production

HEK293T cells were cotransfected with pMD2.G, psPAX2, and the pTRIPZ-mEmerald-PLpro/PLpro^mut^ constructs. The cells were cultured overnight in a complete medium, after which the medium was refreshed, and the cells were cultured for an additional 48 h to allow virus production. The virus-containing culture supernatant was briefly centrifuged and passed through a 0.45 µm filter to remove cell debris before aliquoting and storing at −80°C until use.

### Cell lines and transfection procedure

Early passages of the HeLa, HEK293T, U2OS, and HCT116 cell lines purchased from ATCC were cultured in Dulbecco’s minimal essential medium (DMEM), supplemented with 10% FCS and 10 μg/ml ciprofloxacin (complete medium), and maintained in a 37°C incubator in 5% CO_2_. The cells were transiently transfected using jetOPTIMUS® DNA transfection reagents according to the protocols recommended by the manufacturers. The transfection protocols were optimized for each cell line to achieve at least 50% transfection efficiency. U2OS sublines expressing catalytically active and mutant PLpro under the control of a tetracycline-regulated promoter were produced by lentivirus transduction. Briefly, semiconfluent monolayers of U2OS cells were infected with the lentivirus-containing culture supernatant for 48 h in the presence of 8 µg/ml polybrene, followed by selection in a complete medium supplemented with 2 μg/ml puromycin. Expression of the transduced protein was induced by culture in the presence 3 µg/ml of doxycycline for 24 or 48 h. A stable HCT116 subline expressing the TetOn-mCherry-eGFP-RAMP4 reticulophagy reporter (HCT116-EATR) was previously described [64].

### Tandem mass spectrometry and bioinformatics analysis

Five 10-cm dishes of U2OS cells were transfected with ~10 μg FLAG-PLpro, FLAG-PLpro^mut,^ or FLAG-ev. The next day, the cells were lysed in NP-40 lysis buffer (50 mM Tris-HCl, pH 7.6, 150 mM NaCl, 5 mM MgCl_2_, 1 mM EDTA, 1% Igepal/rCA-630, 10% glycerol) supplemented with protease inhibitor cocktail, 20 mM N-ethylmaleimide (NEM) and 20 mM Iodoacetamide. Ten mg of clarified lysates were mixed with 100 μl of a 50% slurry of anti-FLAG-conjugated agarose gel and incubated overnight at 4°C. After four times washing with NP-40 lysis buffer, bound proteins were eluted with 4X loading buffer and separated by NuPAGE 4–12% Bis-Tris gradient gel electrophoresis. The gels were trypsinized and analyzed by Nano liquid chromatography-tandem mass spectrometry (LC-MS/MS) at the Proteomics Mass Spectrometry facility at the Karolinska Institutet, Stockholm. Proteins that were either absent in four replicas of the FLAG-ev or enriched by Log_2_ ≥2, and that were detected by an average of ≥ 2 unique spectral counts in either or both the FLAG-PLpro and FLAG-PLpro^mut^ immunoprecipitates were considered positive hits. Functional interaction network analysis was performed using the Search Tool for the Retrieval of Interacting Genes (STRING) database v. 12.0 and ToppCluster [98]. STRING integrates information on physical interactions and functional relationships identified by high-throughput biochemical analysis, mining of databases and literature, and prediction from genomic context analysis into Protein-Protein-Interaction (PPI) networks. The functional annotation clustering tool of the Universal Protein Resource (UniProt) was used to identify the overrepresentation of genes in particular functional categories and pathways databases, including Gene Ontology and the Kyoto Encyclopedia of Genes and Genomes (KEGG). Protein interactions were visualized using Cytoscape v. 3.10.1.

### Immunoblotting and co-immunoprecipitation

For immunoblotting, the cells were incubated for 30 min on ice in NP-40 lysis buffer supplemented with protease inhibitor cocktail. For ubiquitination analysis, the lysis buffer was supplemented with 5 M NEM to inhibit DUB activity. After centrifugation at 20,000 × g for 30 min at 4°C, the protein concentration of the supernatants was measured using the Bio-Rad DC protein assay. Equal amounts of lysates were fractionated in NuPAGE 4–12% Bis-Tris gradient gel electrophoresis. After transfer to PVDF membranes, the blots were blocked in 1% PVP-40 solution containing 0.05% Tween-20. The membranes were incubated with the primary antibodies diluted in Tris-buffered saline (TBS) containing 0.1% Tween-20 and 5% nonfat milk for 1 h at room temperature or overnight at 4°C, followed by washing and incubation for 1 h with the appropriate horseradish peroxidase-conjugated secondary antibodies. The immunocomplexes were visualized by enhanced chemiluminescence. For immunoprecipitation and coimmunoprecipitation, the cells were harvested 24 h after transfection and lysed in NP-40-IP lysis buffer supplemented with protease inhibitor cocktail for 30 min on ice followed by capture with 50 µl of anti-FLAG or 20 µl anti-MYC conjugated agarose affinity gel for 4 h or overnight, at 4°C with rotation and washed with IP washing buffer (10 mM Tris-HCl pH 7.6, 150 mM NaCl, 0.5 mM EDTA, 0.5% Igepal/rCA-630 and protease inhibitor cocktail). For immunoprecipitation of ^E^CALR (calreticulin)-GFP, the cell lysates were incubated for 2 h with GFP antibody, followed by capture of the immunocomplexes with protein-G coupled Sepharose beads for 2 h. The beads were washed with lysis buffer, and the immunocomplexes were eluted by boiling in 4×NuPAGE LDS Sample Buffer supplemented with a sample-reducing agent. All images were acquired using a ChemiDoc Imaging system (Bio-Rad), and the intensity of target bands was quantified with the ImageLab software.

### N-degron reporter assay

The N-degron reporters were cotransfected in HEK293T cells with FLAG-ev/PLpro/PLpro^mut^ for 24 h, after which the cells were lysed in NP-40 lysis buffer, and protein expression was assessed by western blot. Where indicated, 100 nM epoxomicin or 100 nM bafilomycin A_1_ (Baf A1) was added to the cultures 16 h before harvesting to inhibit proteasome-dependent degradation to inhibit proteasomal or lysosomal-dependent degradation, respectively.

### Activity-based probes (ABP) labeling

The cells were lysed for 30 min on ice in native NP-40 lysis buffer (50 mM Tris-HCl, pH 7.6, 5 mM MgCl_2_, 250 mM sucrose, 0.5 mM DTT, 1% Igepal/rCA-630) supplemented with protease inhibitor cocktail. Labeling with activity-based probes was performed in a volume of 30 μL in reaction buffer (50 mM Tris-HCl, pH 7.6, 5 mM MgCl_2_, 250 mM sucrose, 5 mM DTT and 6 mM CaCl_2_) containing 30 μg of cell lysates expressing FLAG-ev/PLpro/PLpro^mut^ and 1.5 μM Ub-Vinyl Sulfone (Ub-VS) or 1.0 μM ISG15-Vinyl Pentynyl Sulfone (ISG15-VPS). The reactions were incubated at 37°C for 60 min, terminated by the addition of 4× NuPAGE LDS Sample Buffer, and fractionated in 4–12% Bis-Tris NuPAGE gels.

### Confocal immunofluorescence microscopy

For immunofluorescence microscopy, 0.6–1 × 10^6^ cells were plated on cover slides in 6-well plates and cultured for 24 h before transfection. Semiconfluent monolayers were transfected as described and cultured for an additional 24 h. The cells were then fixed in PBS (Sigma-Aldrich, P4417) 4% paraformaldehyde for 20 min, followed by permeabilization for 20 min in PBS containing 0.1% Triton X-100, blocking for 60 min in PBS containing 2% bovine serum albumin (BSA), and incubation with the indicated dilutions of the primary antibodies for 1 h at room temperature. After washing 3 × 5 min with PBS supplemented with 0.1% Tween-20 (PBST), the cells were incubated for another 1 h with the appropriate Alexa Fluor-conjugated secondary antibodies. The nuclei were stained with 2.5 μg/ml DAPI, and the cover slides were mounted cell side down on object glasses with Mowiol containing 50 μg/ml 1,4-diazabicyclo[2.2.2]octane (DABCO; Sigma-Aldrich, D2522) or ProLong™ Glass Antifade Mountant (Thermo Fisher Scientific, P36982) as anti-fading agent. Images were acquired using Zeiss LSM900-Airy and Leica Stellaris 5X scanning fluorescence confocal laser microscopes. Images were analyzed with the Fiji software. The number and area of SQSTM1/p62 aggregates were obtained with the analyze-particle function.

### ER Autophagy Tandem Reporter (EATR) assay

The expression of the EATR reporter was induced in HCT116-EATR cells grown on cover slides by treatment for 24 h with 2 μg/ml doxycycline before FLAG-PLpro/PLpro^mut^ transfection. After culture for an additional 24 h, the cells culture medium was removed by repeated PBS washing, and the cells were starved by culture for 16 h in Earl’s balanced salt solution (EBSS) medium. The cells were then fixed and stained with the anti-FLAG antibody as described. Images were acquired using a Zeiss LSM900-Airy confocal microscope, and the number of red dots in ≥60 PLpro/PLpro^mut^-positive or -negative cells was counted manually.

### Statistical analysis

Plotting and statistical tests were conducted with data obtained in three or more independent experiments using Microsoft Excel and GraphPad Prism 10. No assumptions about data normality were made, and a two-tailed unpaired Student’s t-test was used to determine statistical significance. The numerical *p* values are indicated in the figures.

## Supplementary Material

Ayala et al Supplementary Information R4.docx

## Abbreviations:

ATE1, arginyltransferase 1; ATG, autophagy related; COVID-19, coronavirus disease 2019; DUB, deubiquitinase; HSPA5/BiP/GRP78, heat shock protein family A (Hsp70) member 5; MAP1LC3B/LC3, microtubule associated protein 1 light chain 3; N-degron, N-terminal degradation signal; Nsp, non-structural protein; PLpro, papain-like protease; PtdIns3K, phosphatidylinositol 3-kinase; PtdIns3P, phosphatidylinositol-3-phosphate; SARS-CoV-2, severe acute respiratory syndrome coronavirus-2; SQSTM1/p62, sequestosome 1; UFD, ubiquitin fusion degradation; ULK1, unc-51 like autophagy activating kinase 1; WIPI, WD repeat domain, phosphoinositide interacting
