# Supplementary material for: Regulation of N-degron recognin-mediated autophagy by the SARS-CoV-2 PLpro ubiquitin deconjugase
Source: Autophagy. 2024 Dec 26;21(5):1019–38. doi: 10.1080/15548627.2024.2442849 (PMC12013424; doi:10.1080/15548627.2024.2442849)
Supplement: Ayala et al Supplementary Information R4.docx [file KAUP_A_2442849_SM5990.docx]

**SUPPLEMENTARY INFORMATION**

|  | | | |
| --- | --- | --- | --- |
| **Table S1.** Annotated functions of the PLpro interacting ubiquitin ligases identified by co-immunoprecipitation and mass spectrometry. | | | |
| **Protein** | **Unique peptides** | **% Coverage** | **Annotated function (UniProt manually curated)** |
| **ZNF598** | 3 | 4.3 | E3 ubiquitin-protein ligase that plays a key role in the ribosome quality control (RQC) that is activated when a ribosome stalled during translation. Ubiquitinates the 40S ribosomal protein RPS10/eS10, RPS20/uS10 and RPS3/uS3, which leads to ribosome disassembly [1]. |
| **LTN1** | 6 | 4.2 | E3 ubiquitin-protein ligase. Component of the ribosome quality control complex (RQC) that mediates the ubiquitination and extraction from the ribosome of incompletely synthesized nascent chains for proteasomal degradation [2]. |
| **TRIM25** | 10 | 18.9 | Functions as a ubiquitin E3 ligase and as an ISG15 E3 ligase [3]. Mediates K63-linked polyubiquitination of the RIGI N-terminal CARD-like region and plays a role in signal transduction that leads to the production of interferons [4]. Promotes the ISGylation of SFN/14-3-3 sigma, an adapter protein implicated in the regulation of several signaling pathway [5]. |
| **TRIM28** | 7 | 12.5 | Tripartite motif protein 28 with SUMO and ubiquitin E3 ligase activity. Negatively regulates aggresome formation [6]. Inhibits autophagy by promoting the degradation of AMPK [7]. Inhibits IFN responses by promoting the ubiquitination and degradation of MAVS [8]. |
| **TRIM33** | 2 | 2.6 | E3 ubiquitin-protein ligase. Promotes SMAD4 ubiquitination and degradation [9]. Mono-ubiquitination of SMAD4 hampers its ability to form a stable complex with activated SMAD2-SMAD3 resulting in inhibition of TGFB-BMP signaling cascade [10]. |
| **RNF213** | 6 | 1.4 | Atypical E3 ubiquitin ligase that can catalyze K63-linked ubiquitination of proteins and lipids. Is involved in lipid metabolism, angiogenesis and cell-autonomous immunity. Restricts the proliferation of cytosolic bacteria by ubiquitinating of the lipid A moiety of bacterial lipopolysaccharide/LPS [11]. Regulates fat storage and lipid droplet formation and inhibits the desaturation of fatty acids [12]. Mediates the ubiquitination and degradation of FLNA (filamin A) and NFAT1, leading to inhibit the non-canonical WNT signaling pathway [13]. |
| **NOSIP** | 2 | 8.8 | E3 ubiquitin-protein ligase that is essential for proper development of the forebrain, the eye, and the face. Negatively regulates nitric oxide production by inducing NOS1 and NOS3 translocation to actin cytoskeleton and inhibiting their enzymatic activity [14,15]. |
| **HERC2** | 11 | 3.2 | E3 ubiquitin-protein ligase that regulates ubiquitin-dependent retention of repair proteins on damaged chromosomes. By controlling the steady-state expression of the IGF1R receptor, indirectly regulates the insulin-like growth factor receptor signaling pathway [16]. Ubiquitinates RAF1/C-RAF and regulates MAP2K3/MKK3-MAPK/p38 signaling [17]. |
| **UBE4B** | 3 | 4.6 | Ubiquitin-protein ligase that may function as an E4 mediating the assembly of polyubiquitin chains on substrates ubiquitinated by another E3 ubiquitin ligase [18]. |
| **UBR4** | 27 | 7.5 | E3 ubiquitin-protein ligase which is a component of the N-end rule pathway [19]. Together with clathrin, forms meshwork structures involved in membrane morphogenesis and cytoskeletal organization [20]. Mediates ubiquitination and proteasomal degradation of ACLY, the primary enzyme responsible for the synthesis of cytosolic acetyl-CoA used in biosynthetic pathways, including lipogenesis and cholesterogenesis [21]. Required for the biogenesis of early endosomes [22]. Co-opted by Dengue virus to degrade STAT2 [23]. |
| **UBR5** | 9 | 4.5 | E3 ubiquitin-protein ligase which is a component of the N-end rule pathway. Downregulates the levels of TRAF3 (TNF receptor associated factor 3), a key component of TLR (toll like receptor) signaling, via the miRNA pathway [24]. Acts as an antiviral factor for MERS-CoV [25]. |

REFERENCES

[1] Juszkiewicz S, Hegde RS. Initiation of Quality Control during Poly(A) Translation Requires Site-Specific Ribosome Ubiquitination. Mol Cell. 2017 Feb 16;65(4):743-750 e4.

[2] Shao S, von der Malsburg K, Hegde RS. Listerin-dependent nascent protein ubiquitination relies on ribosome subunit dissociation. Mol Cell. 2013 Jun 6;50(5):637-48.

[3] Zou W, Zhang DE. The interferon-inducible ubiquitin-protein isopeptide ligase (E3) EFP also functions as an ISG15 E3 ligase. J Biol Chem. 2006 Feb 17;281(7):3989-94.

[4] Gack MU, Shin YC, Joo CH, et al. TRIM25 RING-finger E3 ubiquitin ligase is essential for RIG-I-mediated antiviral activity. Nature. 2007 Apr 19;446(7138):916-920.

[5] Nakasato N, Ikeda K, Urano T, et al. A ubiquitin E3 ligase Efp is up-regulated by interferons and conjugated with ISG15. Biochem Biophys Res Commun. 2006 Dec 15;351(2):540-6.

[6] Chang J, Hwang HJ, Kim B, et al. TRIM28 functions as a negative regulator of aggresome formation. Autophagy. 2021 Dec;17(12):4231-4248.

[7] Pineda CT, Potts PR. Oncogenic MAGEA-TRIM28 ubiquitin ligase downregulates autophagy by ubiquitinating and degrading AMPK in cancer. Autophagy. 2015;11(5):844-6.

[8] Chen YY, Ran XH, Ni RZ, et al. TRIM28 negatively regulates the RLR signaling pathway by targeting MAVS for degradation via K48-linked polyubiquitination. J Biol Chem. 2023 May;299(5):104660.

[9] Dupont S, Mamidi A, Cordenonsi M, et al. FAM/USP9x, a deubiquitinating enzyme essential for TGFbeta signaling, controls Smad4 monoubiquitination. Cell. 2009 Jan 9;136(1):123-35.

[10] Quere R, Saint-Paul L, Carmignac V, et al. Tif1gamma regulates the TGF-beta1 receptor and promotes physiological aging of hematopoietic stem cells. Proc Natl Acad Sci U S A. 2014 Jul 22;111(29):10592-7.

[11] Otten EG, Werner E, Crespillo-Casado A, et al. Ubiquitylation of lipopolysaccharide by RNF213 during bacterial infection. Nature. 2021 Jun;594(7861):111-116.

[12] Sugihara M, Morito D, Ainuki S, et al. The AAA+ ATPase/ubiquitin ligase mysterin stabilizes cytoplasmic lipid droplets. J Cell Biol. 2019 Mar 4;218(3):949-960.

[13] Scholz B, Korn C, Wojtarowicz J, et al. Endothelial RSPO3 Controls Vascular Stability and Pruning through Non-canonical WNT/Ca(2+)/NFAT Signaling. Dev Cell. 2016 Jan 11;36(1):79-93.

[14] Dedio J, Konig P, Wohlfart P, et al. NOSIP, a novel modulator of endothelial nitric oxide synthase activity. FASEB J. 2001 Jan;15(1):79-89.

[15] Schleicher M, Brundin F, Gross S, et al. Cell cycle-regulated inactivation of endothelial NO synthase through NOSIP-dependent targeting to the cytoskeleton. Mol Cell Biol. 2005 Sep;25(18):8251-8.

[16] Osorio FG, Freije JM, Lopez-Otin C. The novel tumor suppressor AIRAPL regulates IGF1R proteostasis. Cell Cycle. 2016;15(7):873-4.

[17] Sala-Gaston J, Pedrazza L, Ramirez J, et al. HERC2 deficiency activates C-RAF/MKK3/p38 signalling pathway altering the cellular response to oxidative stress. Cell Mol Life Sci. 2022 Oct 14;79(11):548.

[18] Koegl M, Hoppe T, Schlenker S, et al. A novel ubiquitination factor, E4, is involved in multiubiquitin chain assembly. Cell. 1999 Mar 5;96(5):635-44.

[19] Tasaki T, Mulder LC, Iwamatsu A, et al. A family of mammalian E3 ubiquitin ligases that contain the UBR box motif and recognize N-degrons. Mol Cell Biol. 2005 Aug;25(16):7120-36.

[20] Nakatani Y, Konishi H, Vassilev A, et al. p600, a unique protein required for membrane morphogenesis and cell survival. Proc Natl Acad Sci U S A. 2005 Oct 18;102(42):15093-8.

[21] Lin R, Tao R, Gao X, et al. Acetylation stabilizes ATP-citrate lyase to promote lipid biosynthesis and tumor growth. Mol Cell. 2013 Aug 22;51(4):506-518.

[22] Kim ST, Lee YJ, Tasaki T, et al. The N-recognin UBR4 of the N-end rule pathway is targeted to and required for the biogenesis of the early endosome. J Cell Sci. 2018 Sep 10;131(17).

[23] Morrison J, Laurent-Rolle M, Maestre AM, et al. Dengue virus co-opts UBR4 to degrade STAT2 and antagonize type I interferon signaling. PLoS Pathog. 2013 Mar;9(3):e1003265.

[24] Cho JH, Kim SA, Seo YS, et al. The p90 ribosomal S6 kinase-UBR5 pathway controls Toll-like receptor signaling via miRNA-induced translational inhibition of tumor necrosis factor receptor-associated factor 3. J Biol Chem. 2017 Jul 14;292(28):11804-11814.

[25] Zhou Y, Zheng R, Liu D, et al. UBR5 Acts as an Antiviral Host Factor against MERS-CoV via Promoting Ubiquitination and Degradation of ORF4b. J Virol. 2022 Sep 14;96(17):e0074122.

[26] Liu J, Nagy N, Ayala-Torres C, et al. Remodeling of the ribosomal quality control and integrated stress response by viral ubiquitin deconjugases. Nat Commun. 2023 Dec 14;14(1):8315.

[27] Dantuma NP, Lindsten K, Glas R, et al. Short-lived green fluorescent proteins for quantifying ubiquitin/proteasome-dependent proteolysis in living cells. Nat Biotechnol. 2000 May;18(5):538-43.

**SUPPLEMENTARY FIGURES**

**TUBA**

**PLpro**


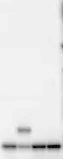

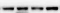

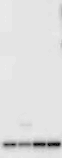

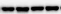


**ISG15/Ub-PLpro**

**+**

**-**

**-**

**-**

**FLAG-PLpro**

**FLAG-PLpro^mut^**

**ISG15-VPS (1.0 µM)**

**+**

**-**

**+**

**-**

**-**

**+**

**-**

**-**

**-**

**+**

**+**

**-**

**+**

**-**

**-**

**-**

**-**

**+**

**-**

**-**

**-**

**+**

**-**

**+**

**Ub-VS (1.5 µM)**

**+**

**-**

**-**

**+**

**B**

**- 50**

**kDa**

**- 150**

**- 75**

**- 50**

**- 37**

**- 250**


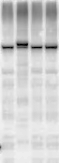

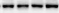

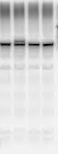

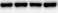


**- 50**

**kDa**

**- 150**

**- 75**

**- 50**

**- 37**

**- 250**

**PLpro-TM**

**+**

**-**

**-**

**-**

**FLAG-PLpro-TM**

**FLAG-PLpro-TM^mut^**

**ISG15-VPS (1.0 µM)**

**+**

**-**

**+**

**-**

**-**

**+**

**-**

**-**

**-**

**+**

**+**

**-**

**+**

**-**

**-**

**-**

**-**

**+**

**-**

**-**

**-**

**+**

**-**

**+**

**Ub-VS (1.5 µM)**

**+**

**-**

**-**

**+**

**C**

**ISG15/Ub-PLpro-TM**

**TUBA**

109

207

387

746

806

1060

1201

1341

1568

1643

1946

1

⍺-helical

746

806

1060

746

806

1060

1201

1341

1568

1643

3x

FLAG

1

330

1

1200

3x

FLAG

–C857

C111–

C111–

1946

**A**

Ubl1

HVR

ADRP

SUD

Ubl2

**PLpro**

NBD

TM

ZnF

Y domain

⍺-helical

Ubl2

**PLpro**

Nsp1

Nsp2

**Nsp3**

Nsp4

Nsp16

**PLpro**

Ubl2

**PLpro**

NBD

TM

ZnF

Y domain

**FLAG-PLpro**

**FLAG-PLpro-TM**

**Figure S1.** Functional validation of PLpro and PLpro-TM constructs in HEK293T cells. (**A**) Schematic illustration of the domain organization of SARS-CoV-2 Nsp3 and PLpro and PLpro-TM constructs used in the study. The position of the catalytic Cys residue relative to the full-length Nsp3 (upper) and catalytic domain constructs (lower) are indicated. For functional validation of the PLpro constructs, HEK293T cells were transiently transfected with FLAG-ev/PLpro/PLpro^mut^ (**B**) or FLAG-ev/PLpro-TM/PLpro-TM^mut^ plasmids (**C**) and cell lysates were labeled with the ISG15-VPS and Ub-VS functional probes. Covalent adducts between the probes and the catalytic Cys residue of the enzyme are visualized in western blots as a migration shift corresponding to the size of the probe. Higher affinity for the ISG15-VPS probe was observed for both the PLpro and PLpro-TM constructs.

**Figure S2.** Cellular PLpro interactome identified by co-immunoprecipitation and mass spectrometry. PLpro interacts with proteins involved in translation, ER stress, and RNA processes. (**A**) Significantly enriched biological processes of the PLpro interacting proteins identified by co-immunoprecipitation and mass spectrometry. U2OS cells were transiently transfected with FLAG-tagged versions of the catalytically active PLpro (FLAG-PLpro, aa 746-1060), or a catalytic mutant where the Cys111 residue was mutated to Ala (FLAG-PLpro^mut^) along with a FLAG-empty vector (FLAG-ev) control. The mass spectrometry data were analyzed as follows: raw data was processed with the Scaffold 5.1.2 software using 95% confidence interval and 2 peptides. The Quantitative Value Normalized Exponentially Modified Protein Abundance Index (emPAI), Exclusive Unique Peptide Count, and Percent Coverage were used for further analysis. The Fold Change was calculated as the ratio between the quantitative values of FLAG-PLpro/PLpro^mut^ and FLAG-ev. Three hundred and thirteen proteins that were identified in four replicates by an average of two unique peptides and were either absent in the FLAG-ev immunoprecipitates or exhibiting a Log2 fold enrichment ≥ 2 are annotated in these processes. Of these, 262 proteins are exclusively found in the FLAG-PLpro immunoprecipitates, 11 were found in the FLAG-PLpro^mut^, and 40 proteins are found in both FLAG-PLpro/PLpro^mut^ immunoprecipitates. (**B**) STRING network diagram of the major protein interaction hubs. The PLpro interactome comprises proteins involved in ribosome biogenesis, mRNA translation, ribosome-associated quality control (RQC), and ER stress responses. Functional clusters are color coded. Proteins listed as PLpro interactors in BioGRID (data base for NSP3 SARS-CoV-2 interacting proteins) are indicated by a purple border. Functionally related proteins that are listed in BioGRID but were not identified in the current analysis are indicated. (**C**) Representative western blots that illustrate the interaction of PLpro with N-recognin ligases. Lysates of HeLa cells transfected with plasmids expressing FLAG-ev/PLpro/PLpro^mut^ were immunoprecipitated with anti-FLAG coated beads, and western blots were probed with the indicated antibodies. GAPDH served as a loading control. Each interaction was validated in at least two independent co-immunoprecipitation experiments.

**Figure S3**. Membrane-anchored PLpro preferentially stabilizes N-degron substrates. (**A**) PLpro-TM selectively stabilizes the N-degron reporter Ub-R-GFP. HEK293T cells transiently cotransfected with the Ub-R-GFP or Ub^G76V^-GFP reporters and FLAG-ev/PLpro-TM/PLpro-TM^mut^. FLAG-ev transfected cells were incubated overnight with 100 nM epoxomicin as a control for ubiquitin-dependent proteasomal degradation. Representative western blots from one out of three independent experiments are shown. (**B**) Densitometric quantification of the GFP-specific bands. The data are displayed as mean ± SD GFP intensity in PLpro-TM/PLpro-TM^mut^ and epoxomicin-treated cells relative to FLAG-ev after normalization to the GAPDH loading control. Significance was calculated by unpaired two-tailed Student’s t-test.


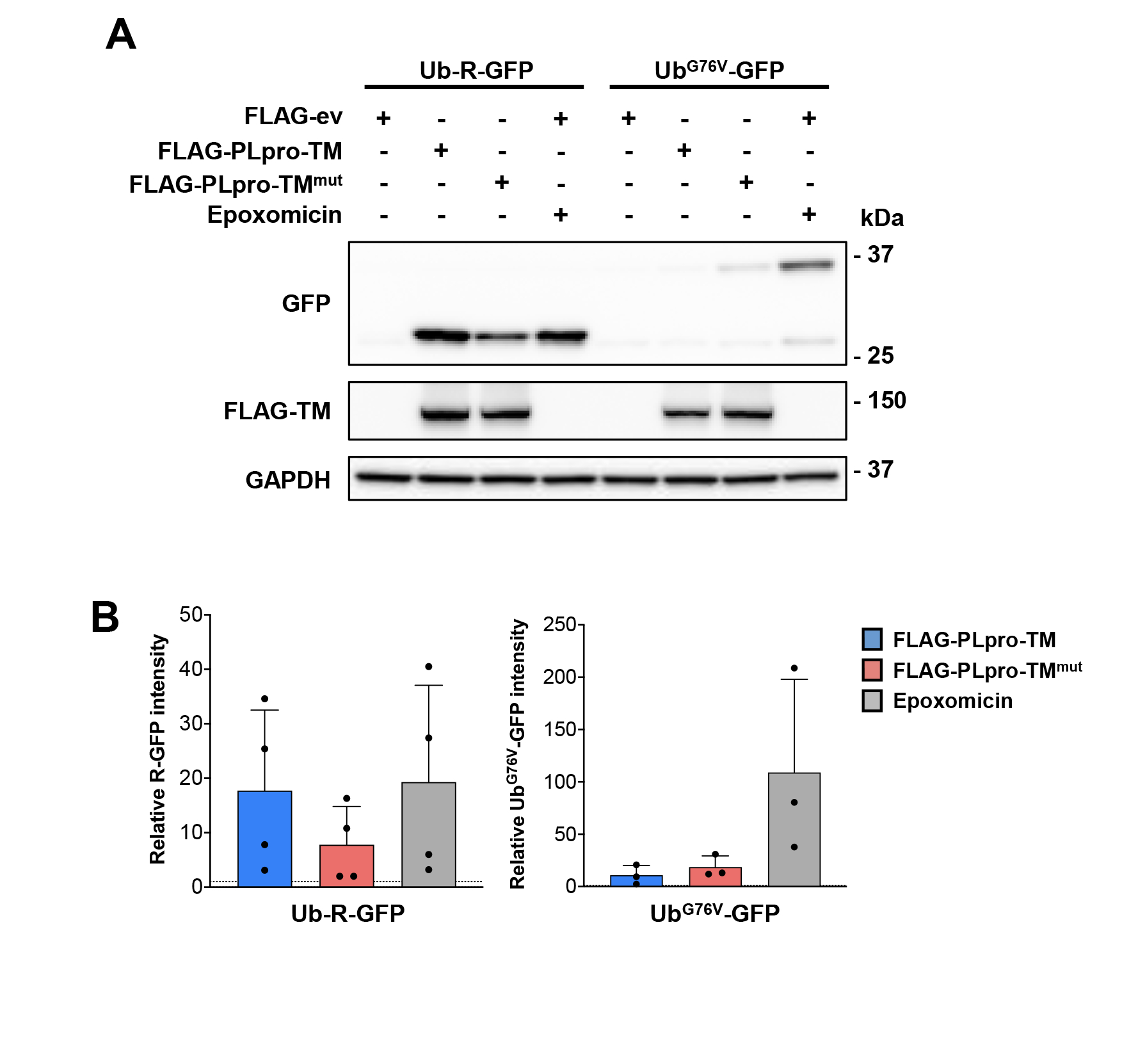


**Ubiquitin**

**- 15**

**- 250**

**- 50**

**- 37**

**- 150**

**- 75**

**- 25**

**- 100**

**MYC-tag**

**kDa**

**- 250**

**- 150**

**- 75**

**- 100**


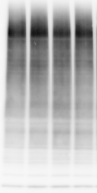

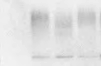


**TUBE IP**

**FLAG-ev**

**+**

**+**

**-**

**-**

**+**

**-**

**+**

**-**

**FLAG-PLpro**

**FLAG-PLpro^mut^**

**+**

**-**

**-**

**+**

**Ub-R-^E^HSPA5-MYC**

**-**

**+**

**-**

**-**

**B**


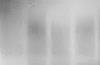


**- 250**

**- 150**

**- 100**


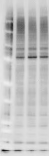


**kDa**

**FLAG-ev**

**+**

**+**

**-**

**-**

**+**

**-**

**+**

**-**

**FLAG-PLpro**

**FLAG-PLpro^mut^**

**+**

**-**

**-**

**+**

**Ub-R-^E^HSPA5-MYC**

**-**

**+**

**-**

**-**

**Ubiquitin**

**MYC IP**

**Input**

**- 15**

**- 250**

**- 50**

**- 37**

**- 150**

**- 75**

**- 25**

**- 100**

**Ubiquitin**

**A**

**Figure S4.** PLpro deubiquitinates R-^E^HSPA5. Aliquots of the cell lysates from HEK293T cells transiently cotransfected with Ub-R-^E^HSPA5-MYC and FLAG-ev/PLpro/PLpro^mut^ used in the blots shown in Figure 4C were immunoprecipitated with anti-MYC antibodies (**A**) or Tandem Ubiquitin Binding Entities (TUBEs) (**B**). For loading controls see Figure 4C. Blots from one representative experiment out of two are shown.

**Figure S5**. Membrane-anchored PLpro stabilizes R-^E^HSPA5. (**A**) PLpro-TM promotes the stabilization of ^E^HSPA5 reporter. HEK293T cells were transiently cotransfected with the FLAG-ev/PLpro-TM/PLpro-TM^mut^ and the Ub-^E^HSPA5-MYC plasmid. As a control for proteasome- and lysosome-dependent degradation, the FLAG-ev transfected cells were treated overnight with 100 nM epoxomicin. The expression of the R-^E^HSPA5 was analyzed 24 h post-transfection by probing western blots with the indicated antibodies. Representative blots from one out of three independent experiments are shown. (**B**) The intensities of the R-^E^HSPA5 bands were quantified by densitometry in three independent experiments. The mean ± SD relative intensity of the R-^E^HSPA5 bands in FLAG-PLpro-TM/PLpro-TM^mut^ transfected or epoxomicin treated cells versus FLAG-ev transfected cells is shown. Significance was calculated by unpaired two-tailed Student’s t-tests.

**B**

**-**

**1**

**3**

**Doxycycline (h)**

**1**

**3**

**6**

**kDa**

**- 50**

**- 37**

**48**

**-**

**6**

**12**

**24**

**48**

**12**

**24**

**Emerald-PLpro**

**Emerald-PLpro^mut^**

**PLpro**

**GAPDH**


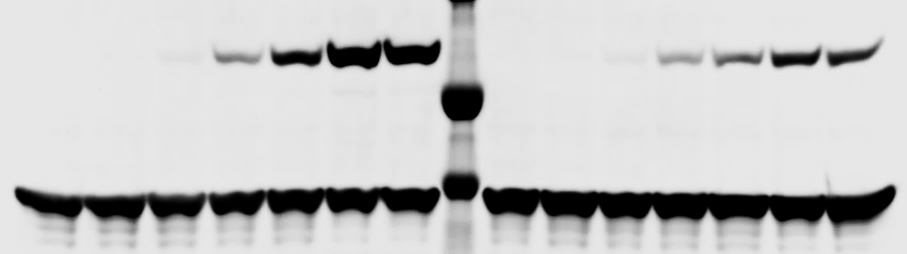


**GAPDH**

**kDa**

**- 37**


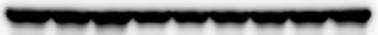

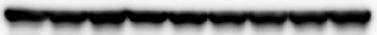

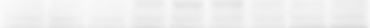


**PLpro**

**GFP**

**- 50**


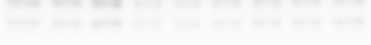

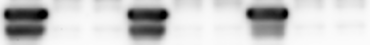


**- 25**


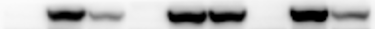


**Emerald-ev**

**+**

**-**

**-**

**-**

**+**

**-**

**Emerald-PLpro**

**Emerald-PLpro^mut^**

**-**

**-**

**+**

**-**

**+**

**-**

**-**

**-**

**+**

**+**

**-**

**-**

**Untreated**

**Epoxomicin**

**C**

**-**

**+**

**-**

**-**

**-**

**+**

**+**

**-**

**-**

**Baf A1**

**-**

**+**

**-**

**-**

**-**

**+**

**+**

**-**

**-**

**Untreated**

**-**

**+**

**-**

**-**

**-**

**+**

**+**

**-**

**-**

**Epoxomicin**

**-**

**+**

**-**

**-**

**-**

**+**

**+**

**-**

**-**

**Baf A1**

**Dox^-^**

**Dox^+^**

**kDa**

**- 75**

**- 37**

**Emerald-ev**

**+**

**-**

**-**

**-**

**-**

**Emerald-PLpro**

**Emerald-PLpro^mut^**

**ISG15-VPS (1.0 µM)**

**+**

**-**

**-**

**+**

**-**

**+**

**-**

**-**

**-**

**+**

**-**

**+**

**-**

**-**

**-**

**-**

**+**

**-**

**+**

**-**

**-**

**+**

**-**

**-**

**+**

**-**

**-**

**+**

**-**

**-**

**-**

**-**

**+**

**+**

**-**

**-**

**-**

**+**

**-**

**+**

**-**

**-**

**-**

**+**

**-**

**-**

**-**

**-**

**-**

**+**

**D**

**Ub-VS (1.5 µM)**


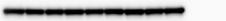


**GAPDH**


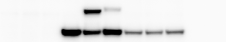

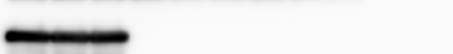


**GFP**

**- 37**

**PLpro**

**ISG15/Ub-PLpro**

**3 µg/ml Doxycycline (48 h)**


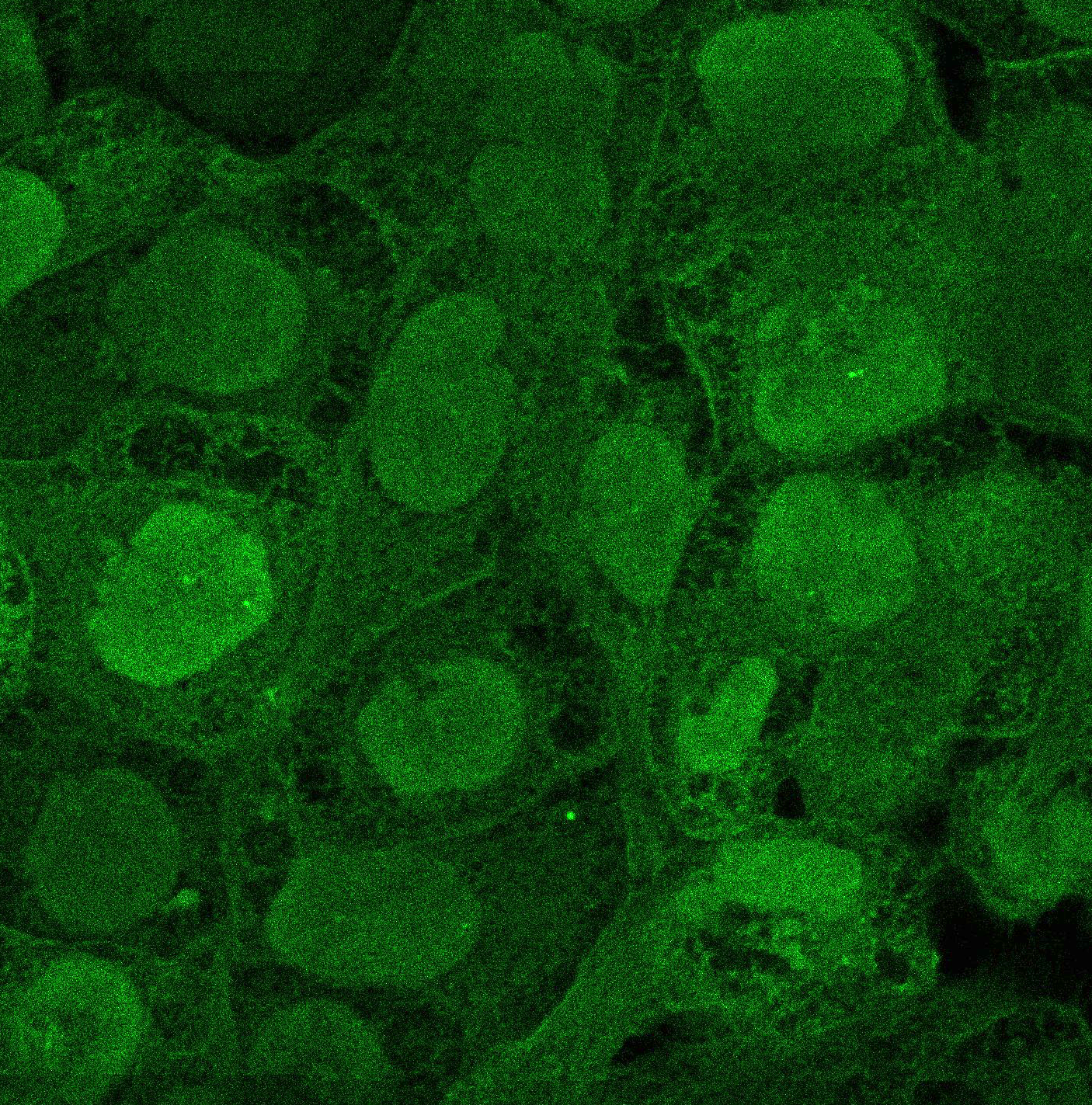

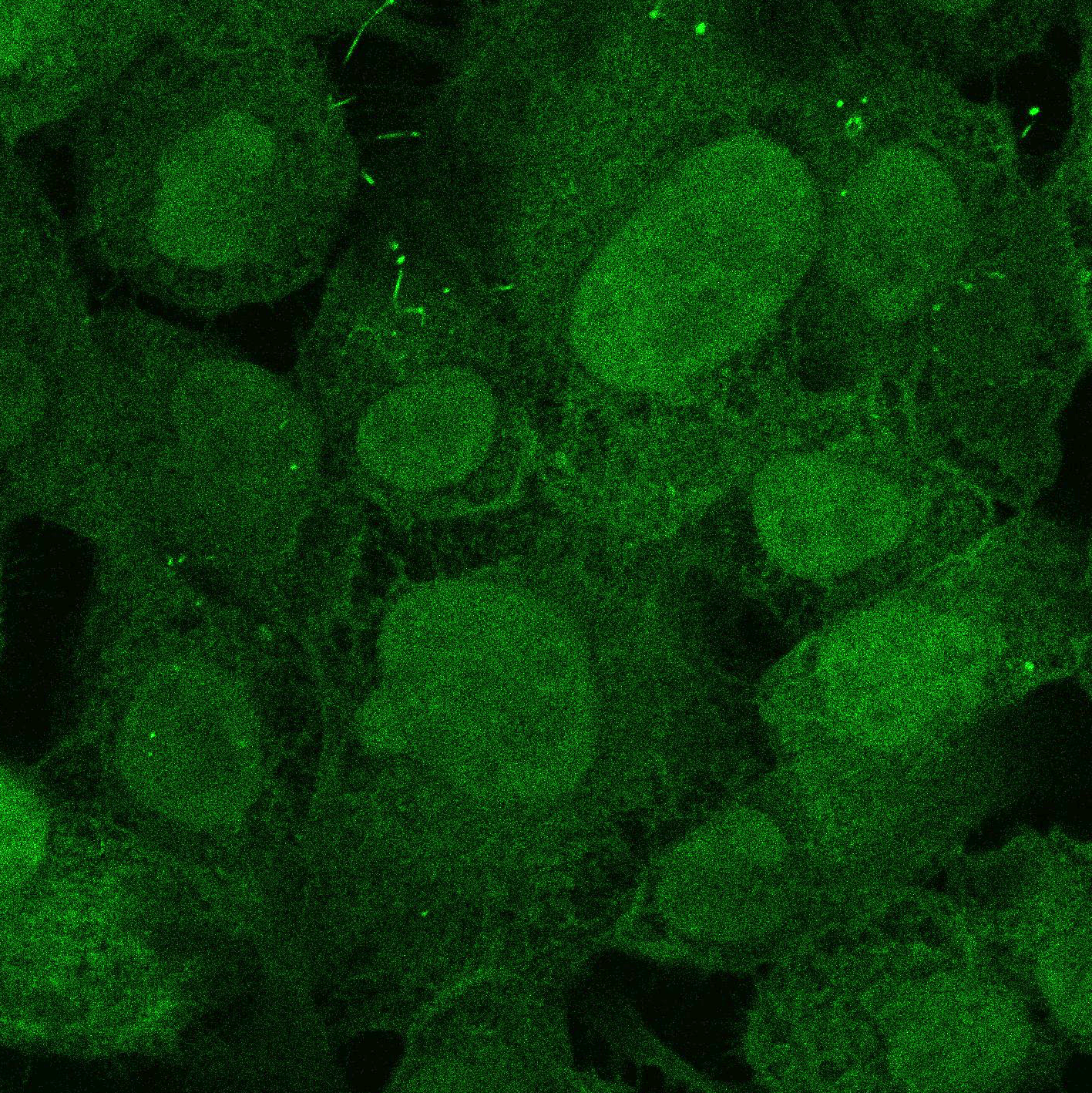

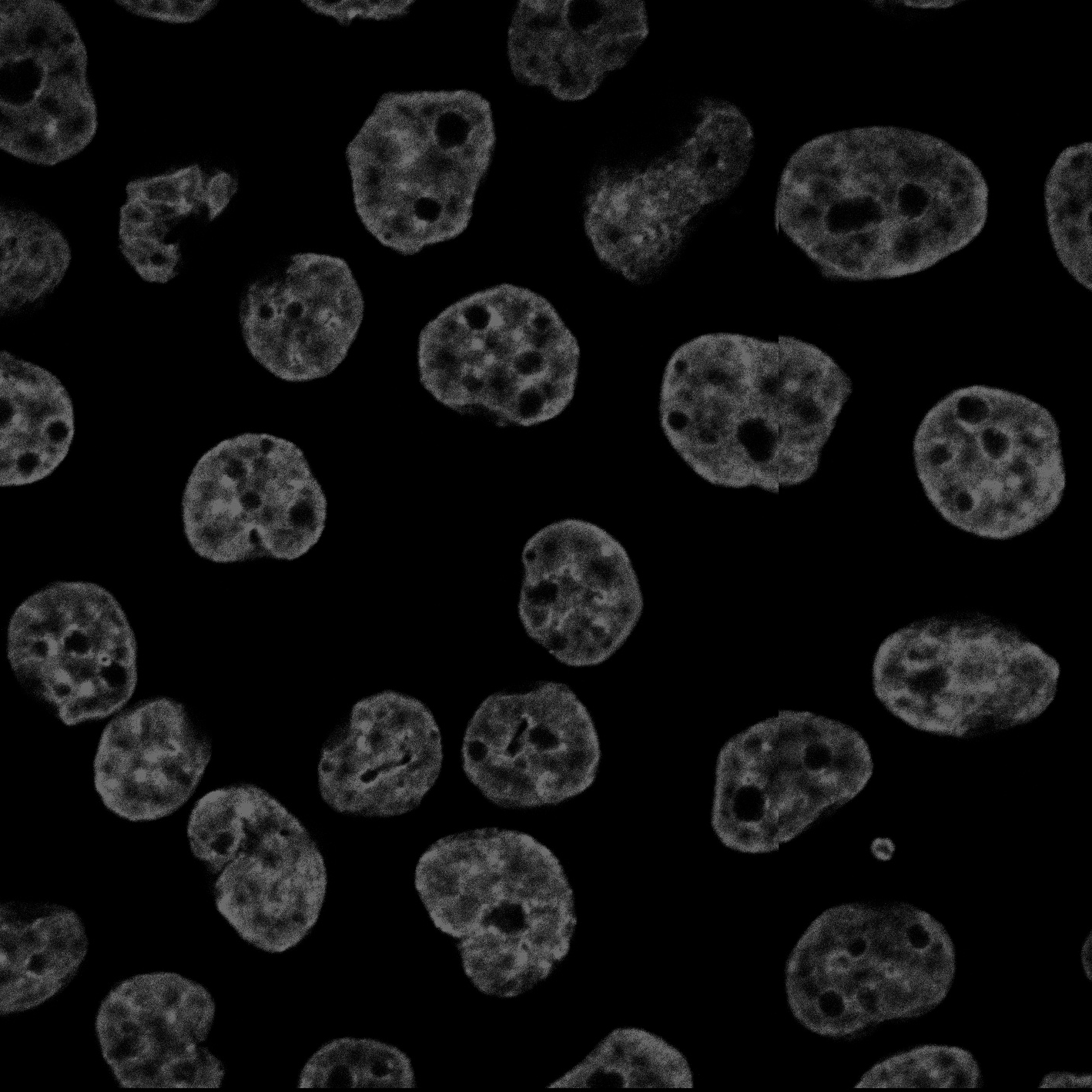


**Dox^-^**

**Dox^+^**


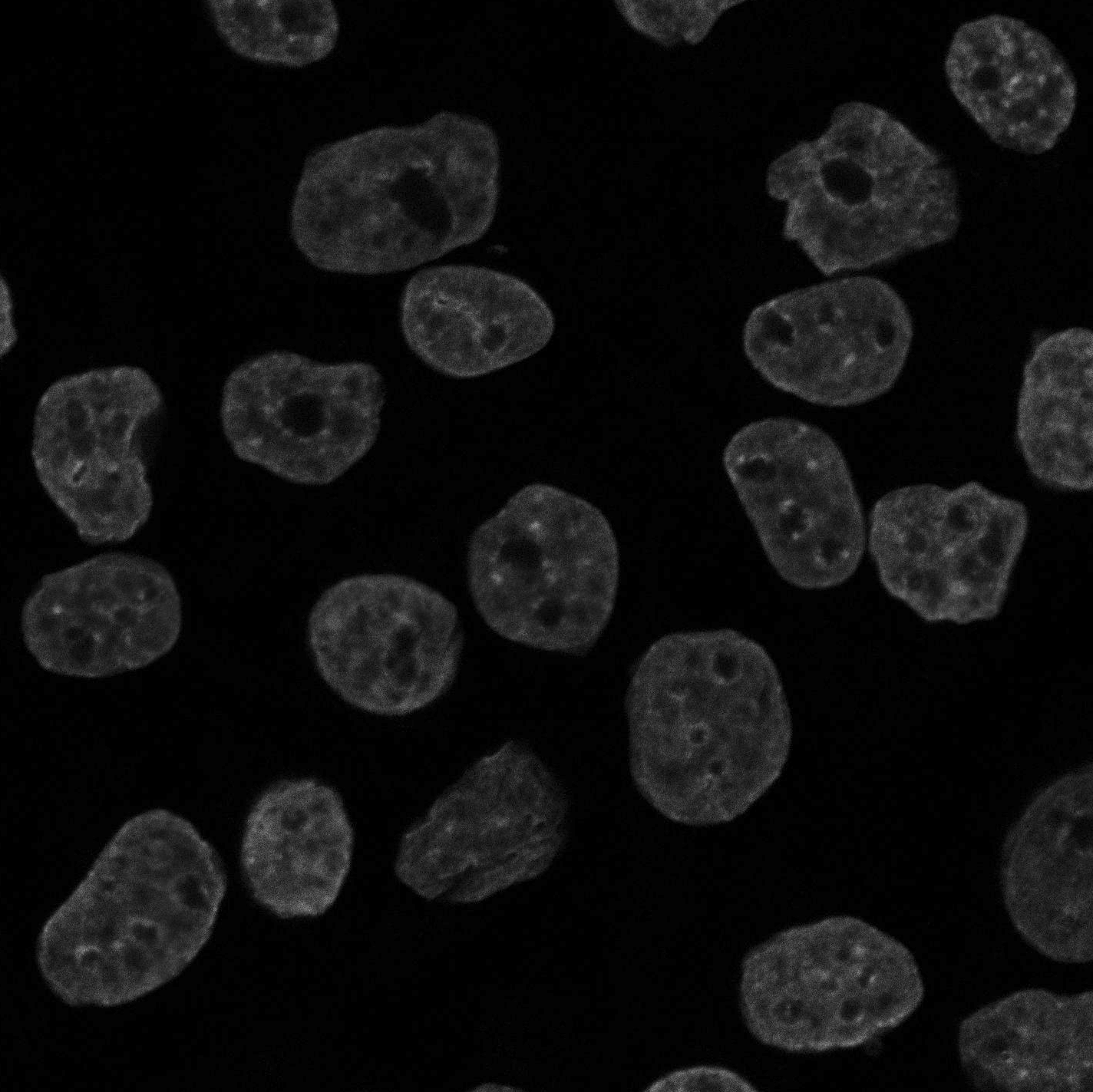


**Emerald-PLpro**

**Emerald-PLpro^mut^**

**A**

**Figure S6.** Characterization of PLpro inducible U2OS cell line. (**A**) Fluorescence images illustrating the expression of Emerald-PLpro/PLpro^mut^ in stably transduced U2OS cells. Scale bar: 10 μm. (**B**) Time kinetics of PLpro/PLpro^mut^ induction in Dox treated cells. Stable U2OS-PLpro/PLpro^mut^-Tet-On cells were induced with 3 μg/ml doxycycline over the indicated times, and the expression of Emerald-PLpro/PLpro^mut^ was analyzed by western blot. (**C**) The PLpro^mut^ polypeptide is degraded by the proteasome. Tet-On U2OS cells were induced in the presence of epoxomicin or Baf A1 and the protein levels of Emerald-PLpro/PLpro^mut^ were analyzed by western blot. (**D)** Functional characterization of the inducible U2OS-PLpro/PLpro^mut^-TetOn cells. Lysates of cells treated overnight with 3 μg/ml doxycycline were incubated for 60 min at 37°C with the ISG15-VPS or Ub-VS probes. Covalent adducts between the probes and the catalytic Cys residue of the enzyme are visualized in western blots as a migration shift corresponding to the size of the probe.

**Figure S7**. Components of the autophagic machinery that are not recruited to PLpro containing complexes. (**A**) FLAG immunoprecipitates of HEK293T cells transfected with FLAG-ev/PLpro/PLpro^mut^ in the presence or absence of co-transfected Ub-R-^E^HSPA5-MYC were probed with the indicated antibodies. The intensity of the ULK1-specific band was strongly decreased in cells expressing catalytically active PLpro, and the protein was not detected in the FLAG-IP. Western blots from one representative experiment out of three are shown in the figure. (**B**) The expression of catalytically active PLpro correlates with reduced expression of ULK1 in transduced U2OS cells. Blots from one representative experiment out of three are shown in the figure. (**C**) Quantification of the ULK1-specific band in three independent experiments. Significance was calculated by unpaired two-tailed Student’s t-tests.
